# Effects of Chemical Fixation on Stimulated Raman Scattering Microscopy

**DOI:** 10.1021/acs.analchem.6c01478

**Published:** 2026-09-23

**Authors:** Chiara Ceconello, Wuji Han, Bryce Manifold, Dario Polli, Aaron Streets

**Affiliations:** † Department of Bioengineering, 1438 University of California Berkeley, Berkeley, California 94720, United States; ‡ Department of Physics, Politecnico di Milano, Piazza Leonardo da Vinci 32, Milano 20133, Italy; § Center for Computational Biology, 1438 University of California Berkeley, Berkeley, California 94720, United States; ∥ CNR-Institute for Photonics and Nanotechnologies (IFN-CNR), Piazza Leonardo Da Vinci 32, Milano 20133, Italy; ⊥ Biophysics Graduate Group, University of California Berkeley, Berkeley, California 94720, United States; # Biohub, San Francisco, California 94158, United States

## Abstract

Stimulated Raman scattering (SRS) microscopy enables label-free imaging of cells and tissues in their native state with chemical specificity. However, there are often experimental advantages of chemical fixation of samples prior to imaging, which can introduce perturbations that may alter the native state of the samples and possibly impact the SRS signal. In this study, we systematically characterize the effects of common fixatives (paraformaldehyde, formalin, glutaraldehyde, methanol, ethanol) on the preservation of cellular integrity and molecular composition in a HeLa cell model as observed by SRS microscopy. We demonstrate how the different fixatives can influence lipid and protein content and overall cell morphology, with significant implications for the accuracy of quantitative SRS microscopy. Our findings indicate that formaldehyde-based fixatives impose minimal disruption to the spatial or spectral features of biomolecules compared to the other fixatives tested and suggest glutaraldehyde (GA) as a suitable alternative for SRS imaging. This study provides insights into the choice of optimal sample preparation, enabling more reliable SRS-based studies for the evaluation of cellular processes and disease mechanisms where fixation is used.

## Graphical abstract

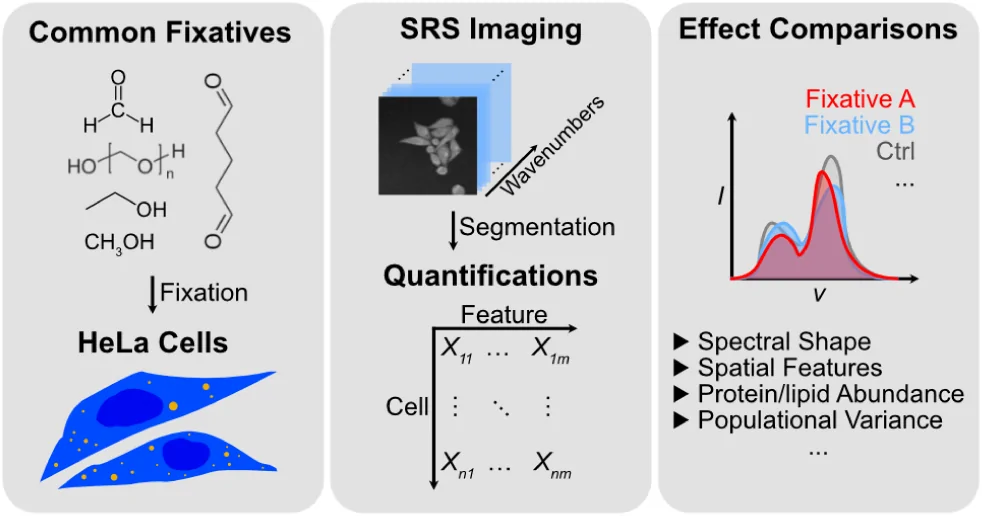

## Introduction

Stimulated Raman Scattering (SRS) is a powerful microscopy technique for label-free investigation of cells and tissues by exciting distinct molecular vibrations to provide unique endogenous signatures for chemical profiling.^1,2^ Offering high-speed quantitative imaging, with high spatial resolution in three dimensions, SRS has demonstrated great potential in metabolic studies and tissue diagnosis^3,4^ without the need for chemical labeling. This allows unperturbed characterization of biological samples, which can be critical to accurately understand biological function in situ. However, while chemical labeling is not required, many SRS studies are performed on fixed samples, calling for preparation protocols to preserve sample characteristics as close to the native state as possible. Chemical fixation, developed in the late 19th century for preserving tissues for microscopic examination,^5,6^ involves adding solvents or cross-linking molecules to samples to “freeze” intracellular molecular content, preventing postmortem decay and providing additional mechanical strength and protection against bacteria.^5,7^ As fixation alters molecular structures, a precise, sensitive, and comprehensive characterization of biomolecular changes is critical for accurate interpretation of SRS images when using fixatives.^8^ Moreover, in experiments requiring the integration of diverse analytical techniques for phenotypic characterization (e.g., SRS microscopy, mass spectrometry, or transcriptomics), understanding compatible fixation methods and their specific effects in cellular chemistry and morphology, ensures clear and accurate results. Vibrational spectroscopy itself, being inherently label-free and chemically specific, is an excellent method for analyzing the effects of fixation on cells.^9–13^ Spontaneous Raman spectroscopy has been used to investigate the effects of sample fixation across various bacteria strains,^14^ eukaryotic tissues,^14,15^ and different human cell lines.^13,16–18^ These fixation-induced changes are characterized spectrally through analysis of key peaks in the fingerprint region of the vibrational spectrum. While spontaneous Raman spectroscopy provides valuable molecular information, its low signal levels make it impractical for fast, high-resolution imaging of cells and tissues. Coherent Raman, particularly in the C–H stretching region, overcomes this limitation by enabling rapid, label-free chemical imaging with high spatial resolution. Techniques like CARS and SRS have proven effective in visualizing cellular content and processes, successfully detecting intracellular lipids, proteins, nucleic acids, and water with high speed and sensitivity.^3,19–23^ Despite the increasing adoption of coherent Raman imaging in biological research,^24–26^ a systematic characterization of how fixation affects these measurements is still lacking. In this work, we address this gap by exploring the effects of common fixatives on SRS microscopy images of biological samples, providing a more comprehensive characterization of fixation-induced changes through high-resolution chemical and morphological features.

Here, we explore the effects of common fixatives on SRS microscopy images of biological samples (Figure 1). We offer insights into both the morphological and chemical effects of these fixatives on HeLa cells, a well-characterized, widely used, and reliable cell line model that serves as an excellent platform for exploring fixation effects. We extend the analysis of cell fixation via a broad range of protocols explored in the C–H stretching region through SRS microspectroscopy, providing additional quantitative morphological insights. We select five commonly employed fixatives, chosen for their widespread adoption in microscopy and their popularity in adjacent fields (e.g., DNA and RNA sequencing), to evaluate against live samples. This study offers a broader characterization of fixation-induced changes in SRS microscopy, aiming to inform the design of experiments that integrate label-free chemical imaging with orthogonal approaches.

**Figure 1:**
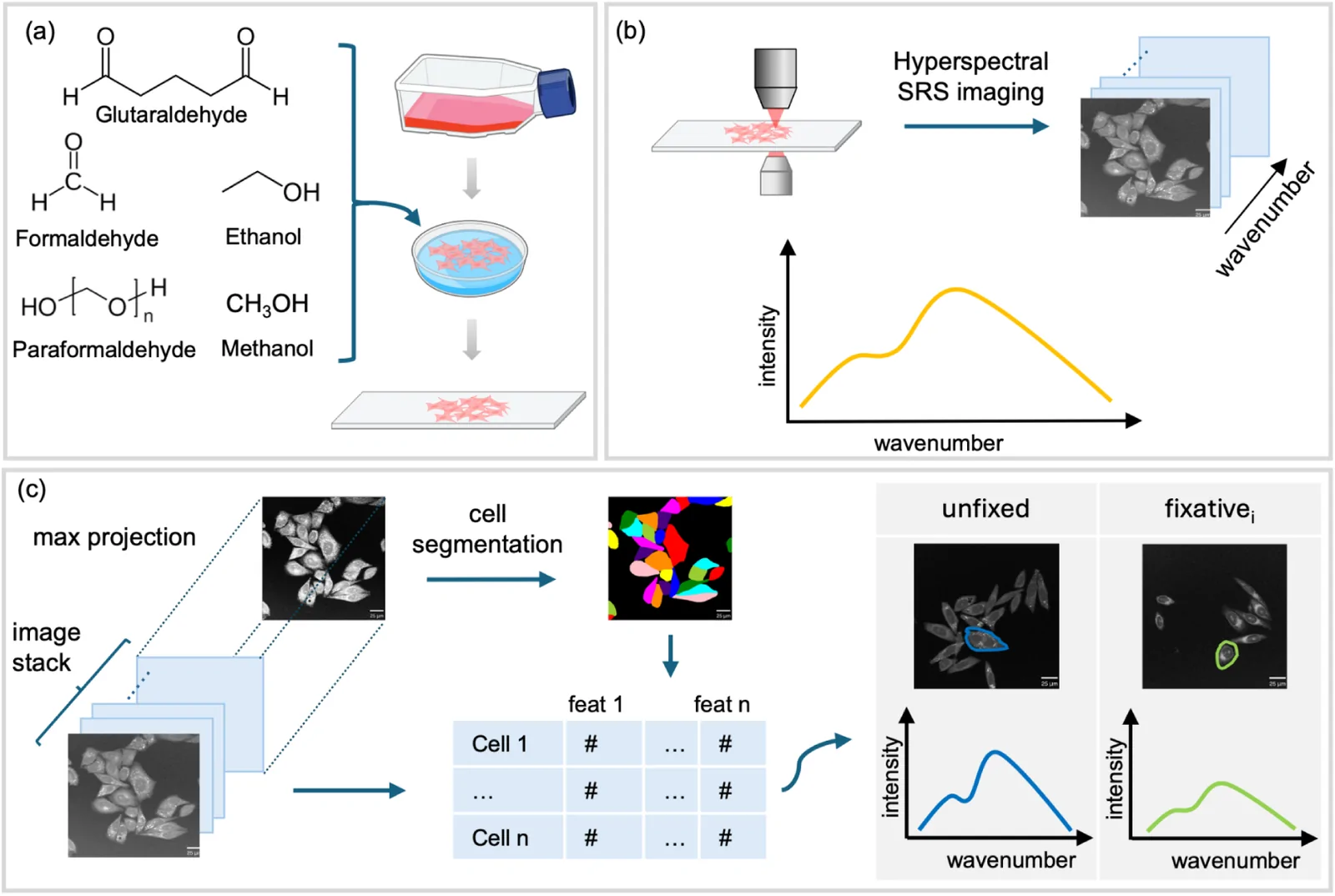
Schematic representation of the fixation SRS experiment. (a) Cell culture, fixation and seeding on microscope slides. (b) Stimulated Raman Scattering data hypercube acquisition in the C–H stretching spectral region. (c) Image post-processing workflow: each hyperstack is preprocessed and segmented to extract single-cell metrics and build a comprehensive Raman shift-specific data frame. Then fixatives are compared to the live counterpart both spatially and spectrally.

We aim to provide supporting evidence that PFA (or other cross-linkers, e.g., NBF) is indeed the least disruptive and recommended choice for coherent Raman microscopy-based assays. We also evaluate the effects of glutaraldehyde and propose it as a suitable alternative for SRS imaging, owing to a potentially nondisruptive signal enhancement.^27^ Finally, we quantify the effects of lipid loss and cell shrinkage resulting from alcohol-based fixation as well as exploring other metrics such as heterogeneity across cells and the spatial distribution of signals.

Commonly used fixatives include aldehydes (formaldehyde and glutaraldehyde), alcohols (methanol and ethanol), ketones (acetone), and acids (tannic acid, picric acid, etc.). When used individually, these fixatives often induce undesirable chemical alterations in biological samples, and it is common to use them in combination to balance out unwanted effects.^17^ The predominant cell fixative employed in biology is formaldehyde, typically used in a polymer form such as paraformaldehyde (PFA) at 4% dilution, in an aqueous solution of formaldehyde gas (37–40% by weight) with a small amount of methanol added to prevent polymerization, or as neutral buffered formalin (NBF) at 10% dilution. It is highly used in SRS microscopy,^24,28–30^ as formaldehyde-based fixatives are thought to preserve major biochemical components of the cell in a form that is closest to that in the live cell.^17^ During fixation, the methylene glycol component of formaldehyde forms cross-linkages between the NH_2_ terminals of protein amino acid residues and the NH group of a tertiary amide.^11^ Fixation in formalin thus adjusts the vibrational freedom of membrane lipids, possibly through a reaction of methylene glycol in aqueous formalin with unsaturated hydrocarbon chains,^9^ causing lipid leaching.^31^ Formaldehyde fixation also disrupts hydrogen bonds within large intracellular molecules^7^ and denatures intracellular proteins by forming cross-links among amine residues,^7,9,32^ leading to the loss of quaternary, tertiary, and possibly secondary structures of proteins.^33^ Formaldehyde fixation has also recently been shown to change intracellular hydrogen-bonding spectra in SRS microscopy, suggesting a significant change in free intracellular water.^34^ Protein cross-linking may cause overall sample shrinkage^7^ and formation of nanoscale structures caused by aggregation of membrane proteins both in formaldehyde and glutaraldehyde fixation.^35^ Moreover, prolonged exposure to formaldehyde reduces antigen-binding specificity in immunohistochemical staining,^33^ decreases DNA solubility, and slowly hydrolyzes the DNA and RNA backbones,^7,36–38^ effectively reducing nucleolar RNA concentration.^8^ Previous work has shown that formalin fixation unstacks DNA bases in adenine-thymine-rich regions, forms hydroxylmethyl groups with nucleic acid bases, and hydrolyzes N-glycosidic bonds, resulting in free purine and pyrimidine groups.^7^ Due to the loss of nuclear integrity,^13^ alcohols are generally preferred to formalin for their better preservation of antigens, despite causing overall cell shrinkage.^39,40^ On the basis of this, we expect formalin-fixed cells to appear smaller when imaging with SRS, due to overall shrinkage from intracellular water loss and to show a potential reduction in Raman scattering in the C–H region due to lipid leaching and disruptions to protein folding and structure.

Glutaraldehyde, typically used in 1.25–2.5% dilutions, is an alternative to formaldehyde-based fixation. It forms covalent cross-links between amine residues in proteins.^14,17^ Compared to formaldehyde, glutaraldehyde penetrates cells and tissues more slowly but exhibits stronger cross-linking due to its two aldehyde groups.^31^ This makes it particularly suitable for electron microscopy, where stronger cross-linkers are preferred.^41,42^ Additionally, like formalin-based fixatives, glutaraldehyde preserves cytoplasmic lipid content^9^ and has been observed via Raman spectroscopy to increase CH_2_ bending and CH_2_/CH_3_ twisting relative to live cases.^27^ Because of its stronger cross-linking causing higher structural rigidity, we expect effects from glutaraldehyde to be less pronounced than formaldehyde in size variation. However, a more pronounced change in contributions from CH_2_ and CH_3_ groups to the Raman spectra could be observed, potentially reflecting closer-to-live levels or higher intensity due to significant structural and chemical rearrangements.

Finally, alcohols such as methanol and ethanol are thought to better preserve nucleic acids but have strong denaturing effects on proteins.^31^ They may solubilize membrane lipids,^43^ causing lipid leaching^44,45^ and overall tissue shrinkage,^8^ which is why they are often combined with tissue-swelling fixatives. Despite causing protein rearrangement favoring β-sheet over α-helical conformations,^46^ the cell shrinkage effect increases concentrations of nucleolar proteins and RNA, making alcohol fixation preferred for RNA-based assays such as transcriptomics.^8^ In single-cell transcriptomics, methanol-fixed samples maintain a cellular composition similar to fresh samples, providing good cell quality with minimal expression biases.^47^ The precipitation of other macromolecules (i.e., aggregation out of solution) occurs over several minutes,^35^ with denatured proteins partially refolding upon rehydration in phosphate-buffered saline (PBS).^46^ We expect SRS images of alcohol-fixed cells to show little to no lipid signal, significant shrinkage due to dehydration, and an overall reduction in protein signal, with higher protein concentration localized in the nuclear region.

## Methods

### Cell Sample Preparation

HeLa cells (UC Berkeley Tissue Culture Facility) were cultivated and maintained in Dulbecco’s Modified Eagle’s Medium (DMEM) (Corning) supplemented with 10% Fetal Bovine Serum (FBS) and 1% PenStrep (Penicillin, Streptomycin, l-glutamine) at 37 °C and 5% CO_2_. In preparation for imaging, cells were plated at 20–30% confluence on 8.8 cm^2^ Petri dishes (Nunclon Delta Surface, Thermo Scientific), each prepared with a No.1 18 × 18 mm micro cover glass (VWR), and allowed 24 h to adhere.

At ∼50% confluence, cells were washed three times with 1× PBS (Corning). For live-cell imaging, the cells plated on a micro cover glass were covered with a microscope slide (VWR Vistavision, glass 1 mm thick) to create a sandwich-like configuration with 20 μL of 1× PBS to prevent drying. Multiple fields of view were acquired within 30–40 min of removal from media to avoid alterations in live cells due to drying.

### Fixation Protocols and Experimental Design

For cell fixation (Figure 1a), five mixtures were prepared as

1. 4% Paraformaldehyde (PFA) solution, pH balanced in PBS (Premixed, Invitrogen Thermo Fisher), stored at 4 °C;
2. 10% Neutral Buffered Formalin (NBF) in PBS (Premixed, G-Biosciences), stored at 4 °C;
3. 1.5% Glutaraldehyde (GA) (25% initial aqueous solution, Thermo Scientific) solution, pH balanced in PBS, stored at 4 °C;
4. 10% Methanol (MeOH) (99.8%, Thermo Scientific) dilution in molecular-grade water, stored at −20 °C;
5. 70% Ethanol (EtOH) (200 proof, molecular-grade, Fisher BioReagents) dilution in molecular-grade water, stored at −20 °C;

Raman spectra of the fixation mixtures in the above percentages are provided in the Supporting Information (Figure S1).

Similar to the live-cell protocol, 3× washes were performed with PBS. 2 mL of mixture were added to the Petri dish and allowed to fix for 15 min at room temperature for cross-linking fixation (PFA, NBF, GA) or 10 min at −20 °C for alcohols (MeOH, EtOH). In all protocols, mixtures were then aspirated and followed by 3× washes with PBS. Fixed cells on the microcover glass were then sandwiched with a microscope slide with 20 μL of 1× PBS to prevent drying, and imaged. This experimental workflow was replicated on different days to control for sample-dependent and day-dependent variations.

### Stimulated Raman Scattering Microscopy

A dual-output InSight DS+ laser operating at an 80-MHz repetition rate emits 100–200 fs pulses at the output: a 1040 nm Stokes beam and a tunable pump set at 799 nm, targeting the C–H stretching spectral region, covering the 2820–3100 cm^–1^ range through spectral focusing SRS.^48,49^ Stimulated Raman loss (SRL) is measured modulating the Stokes beam through an EOM (Thorlabs) at 10.28 MHz. The Stokes power is controlled via a motorized half-wave plate-polarizing beam splitter pair. Both beams are chirped with three H-ZF52 glass rods (Union Optic), reaching ∼2 ps duration. A motorized linear delay stage (Newport FCL200) and broadband retroreflective mirror (Newport) pair adjusts pump-Stokes beam overlap to achieve spectral focusing in the C–H stretching region. A dichroic mirror recombines pump and Stokes beam, then coupled with a fully automated commercial inverted microscope (Olympus IX83). Both excitation beams are focused on the sample, mounted on a motorized XY stage (HLD117 Prior), with a water immersion 60×, NA = 1.2 objective (W/IR UplansApo, FN26.5, Olympus) and collection is via an oil-immersion condenser with NA = 1.4 (D-cuo achr-apl Nikon—CSC1003, Thorlabs). The SRL signal is retrieved by demodulating the pump beam with a lock-in amplifier (HF2LI Zurich Instruments) after being filtered (FESH1000 Thorlabs) and collected with a photodiode (Hamamatsu S3590-08). Prior to imaging, the system is calibrated each day using 20–50 μL of 25% DMSO sandwiched between a microscope slide and coverslip, using a double-sided tape as a spacer. A single-point spectrum is acquired to match the delay line position with known spectral peaks. The two DMSO peaks (2913 and 2994 cm^–1^) were identified with a Gaussian mixture model, and a linear model was used to interpolate Raman transitions (wavenumbers). The spectral resolution of the linear model is typically within 20 ± 1 wavenumbers.

For cell imaging, powers were set to 20/25 mW (Pump/Stokes). For each measurement, hyperspectral images were acquired at 16 spectral points, every 12.62 cm^–1^ corresponding to Raman shifts ranging from 2820 to 3012 cm^–1^, targeting both peaks used for characterizing lipid and protein (Figure 1b). Each acquired field of view (FoV) is 512 by 512 pixels, covering 212 × 212 μm^2^. With the galvo scanner’s pixel dwell time (PDT) set to 8 μs, a typical frame time is around 4 s with initialization and save-time overhead. For each microscope slide, 4–6 FoVs were imaged typically. Every FoV would take 1–2 min for viewfinding and focus adjustment, then an automated script takes over the hyperspectral acquisition by stepping the delay stage. A hyperspectral scan takes 60–80 s, and one sample typically has 2–6 fields of view.

To ensure consistency across different imaging days, all measurements were corrected for the highest lock-in gain. Prior to analysis, the raw image hyperstacks underwent field correction by normalizing with a 25% DMSO calibration solvent image. To be specific, in each experiment, a Gaussian filter with a sigma value of 50 (fwhm = 117.74 pixels to match the DMSO image) was applied on the average of three DMSO solvent images and divided by its maximum value. The pixel-wise inverse of the reference solvent image was used as a relative normalizer for cell sample images in the same experiment. This procedure enhances signal uniformity, particularly at the edges of images, which are typically less intense due to chromatic aberrations introduced by the simultaneous point-scanning of the two beams through the high-NA objective across the field of view (FV12-SU high-sensitivity galvo-scanning unit, part of the Olympus FV1200 microscope system).

### Quantification and Analyses

#### Cell Segmentation and Single-Cell Statistics

To isolate single-cell spectra from the images, we used the pretrained Cellpose-SAM model^50,52^ to perform cell segmentation. A maximum projection of the hyperspectral image stack was first performed to create a 2D image for Cellpose. Then, for each image, a U-Net is used to generate horizontal and vertical gradients, which form a vector field. By grouping together pixels that converge to the same center, we could draw individual cell masks.

After the mask image is created from the output, individual cell labels are used to iterate through summary statistic calculations. For example, the sum of intensities adds up all of the pixel values from the same cell, and the *k*-th *q*-quantile calculation outputs the intensity value based on the cumulative distribution of the intensities.

For each field of view, a table with cell labels as rows and statistical types as columns can be generated. To merge multiple fields of view and experiments, each image gets a unique label based on the concatenation of the experiment label, field-of-view label, and cell label.

For metrics of interest, one-way analysis of variance (ANOVA) tests were performed across fixation conditions. After verifying significance, Tukey’s post hoc tests (HSDs) were performed to compare individual group pairs. Thresholds for (adjusted) p-values were set as follows: 0.05 for significant (one star), 0.01, 0.001, and 0.0001 for two, three, and four stars, respectively.

#### Spectral Similarity Examination via Cross Correlation

Given that spectra across conditions are nearly identical, an unbiased way to compare spectral shape is preferred. This is achieved by calculating the correlations between normalized spectra. The bootstrap test for each condition pair is designed as follows.

First, 200 cells are sampled from each condition. The spectrum for each cell based on average intensities at different Raman shifts was extracted. These spectra were then normalized by dividing their maximum value along the spectral axis (Raman shift) in order to average them into one single spectrum.

A pair of average spectra were used for each condition pair to calculate the Pearson product-moment correlation coefficient, i.e.

$$r=\frac{\sum \left(x_{i}-\mathrm{x̅}\right)\left(y_{i}-\mathrm{y̅}\right)}{\sqrt{\sum {\left(x_{i}-\mathrm{x̅}\right)}^{2}\sum {\left(y_{i}-\mathrm{y̅}\right)}^{2}}}$$

The sampling was then repeated for a total of 100 times. This would yield the mean and standard deviation of *r*.

Since *r* is very close to 1, a log-scale representation was used for visualization, where *s* = −log­(1 – *r*) is used to indicate how close *r* is from 1. Thus, the closer *r* is to 1, the higher the similarity (*s*) between the spectra.

#### Spectral Unmixing and Reconstruction

Component spectra contributions were isolated through unmixing via the N-FINDR algorithm.^26,51^ The dimensionality of the hyperspectral image was reduced by finding *n* pure spectral components that best represent the data. To achieve that, the N-FINDR algorithm optimizes the volume of an s-dimensional simplex in an (*s* + 1)-dimensional space; here, s denotes the spectral dimension. The vertices, i.e., endmembers of the simplex become the “pure spectra” after the optimization, and their relative abundance in each pixel can be reconstructed throughout the whole image, utilizing methods such as nonnegative least-squares fitting.

#### Selection of Representative Cells for Visualization

Using the per-cell statistics described above, a representative cell was selected for each fixative condition to illustrate typical morphology and spectral composition. For each condition, the mean SRS intensity within the cell mask at the 2908 cm^–1^ band was computed across all cells, and the cell whose value lay closest to the condition median was chosen. The corresponding field of view was then retrieved, and rendered as either a maximum intensity projection or a two-color composite images.

## Results

### Single-Cell Analysis Reveals Fixation-Dependent Variation in Raman Spectra

We used SRS microscopy to image the cells, either unfixed or treated with one of the five fixatives, as described above. Each condition includes images taken from 5 to 6 independent experiments and at least 6 biological replicates, details shown in Table S1.

We performed a max projection across the wavenumber axis to provide an estimate of overall signal intensity and used these images to visualize the impact of fixation on cells (Figure 2a–f). With respect to unfixed cells, we observed a higher overall SRS signal for glutaraldehyde-fixed cells, some signal reduction in paraformaldehyde- and formalin-fixed cells, and a more prominent signal change for alcohols.

**Figure 2:**
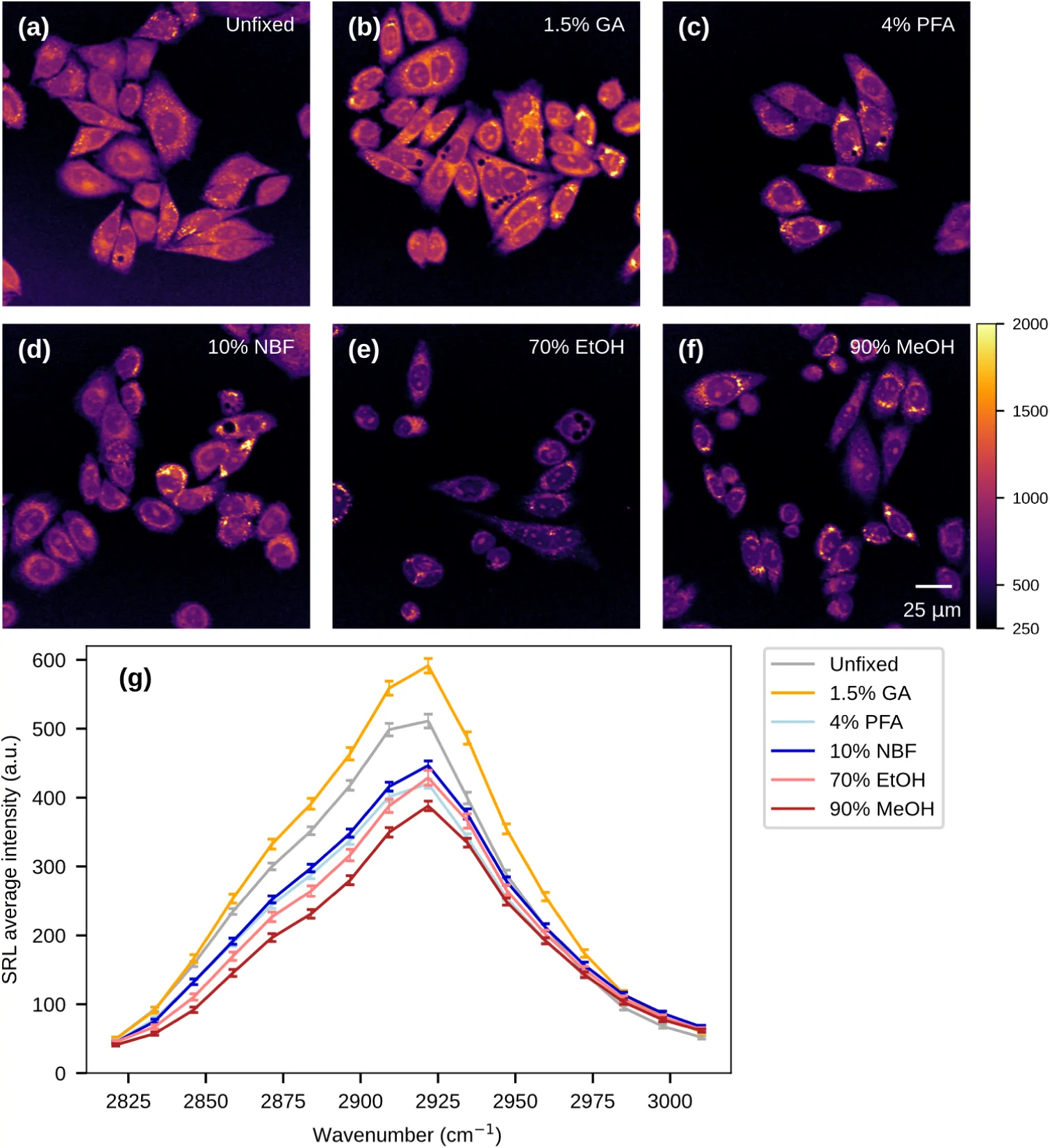
SRS hyperspectral images of fixed and live cells reveal morphological and spectral differences across conditions. (a–f) Maximum projection image over Raman wavenumbers of six representative SRS images, one per fixation condition. (g) Estimated Raman spectra with respect to different fixation conditions. Each point in the lines is the mean of SRS signal intensities of all cells from a certain fixation condition, e.g., mean intensity of all cells from unfixed cells at 2850 cm^–1^. Each vertical error bar represents the bootstrap-estimated (1,000 resamples) 95% confidence interval of the mean for the corresponding fixation condition (color) at that wavenumber (x-position), computed from 308–604 cells pooled across 6–10 biological replicates per condition (Table S1).

We then explored SRS spectra of single cells across all of the conditions. To extract single-cell SRS spectra from the hyperspectral image stacks, we used Cellpose-SAM,^50,52^ a pretrained model for segmentation, to create individual cell masks for single-cell metric extraction. Individual cell masks were segmented with the maximum projection image (see Methods). Each individual cell’s mask was then applied on the hyperspectral stack, creating summary statistics at every Raman shift for each cell. The summary statistics include the sum of SRS intensity, area, and intensities at different pixel quantiles. We constructed a comprehensive data frame of 2325 cells (>300 cells per condition, Table S1) across all experimental conditions and imaging days. We then extracted Raman shift-specific average signal intensity (cumulative cell intensity divided by its size) and cell size across all samples and conditions (Figure 1c).

To estimate a representative Raman spectrum for each fixation condition, the single-cell average SRL intensities above were averaged within each condition (Figure 2g), with the vertical error bars representing a bootstrap-estimated 95% confidence interval of the condition-level mean at each wavenumber. In glutaraldehyde-fixed cell spectra, we observed an overall signal increase with respect to unfixed (16% increase at the 2920 cm^–1^ band, Table S2), consistent with previous reports of Raman signature changes,^27,53^ and our observation on max projection images that appear consistently brighter (Figure 2a). Paraformaldehyde and formalin cause a signal reduction, of respectively, 18% and 16% decrease at the 2920 cm^–1^ band, in agreement with previous works.^8,13,17^ Alcohol fixation results appeared to be the most disruptive, with signal reductions up to 16% (ethanol) and 24% (methanol) at the 2920 cm^–1^ band, coupled with visible cell shrinkage (Figure 2e,f) and internal rearrangement, as reported in the literature.^8^

We calculated the cross-correlation between average spectra from different fixation conditions to further explore how fixatives perturb the chemical composition of the samples. Pearson’s product–moment correlation coefficient (*r*) was computed point by point across the spectra, and transformed as a measure of Similarity *s* = −log­(1 – *r*) to enhance the visibility of small deviations at high similarity values (see Methods). This analysis distinguishes between uniform signal reduction and changes in specific spectral features. As shown in Figure 3a (top row), all fixatives altered the single-cell C–H stretching spectral signature to some extent, with paraformaldehyde and glutaraldehyde showing the highest similarity values to unfixed cells, i.e., 5.3 and 5.2, respectively. While formalin showed a smaller signal reduction than paraformaldehyde in terms of intensity, it exhibited greater spectral distortion (*s* = 4.5). Dissimilarity increases with alcohol fixation, with ethanol showing a similarity value of 3.8 and methanol 3.3, reflecting greater alteration of the spectral profile.

**Figure 3:**
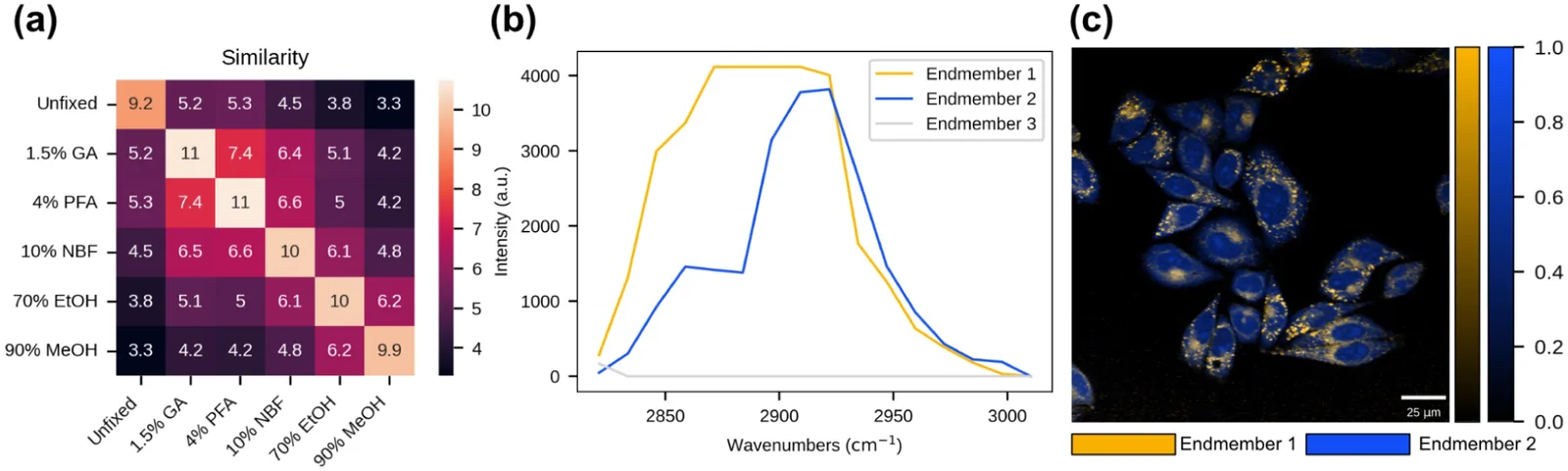
Spectral analysis of hyperspectral SRS images of cells. (a) Spectral similarity assessment by cross-correlation of spectrum pairs. Spectra from individual cells were extracted based on their average intensities at multiple wavenumbers. These spectra are then normalized by dividing their maximum spectral value. Spectra from individual cells were extracted from different fixative conditions. These spectra were then resampled per condition to perform a bootstrapping Pearson product-moment correlation coefficient calculation, between condition pairs. The similarities are calculated with −log­(1 – *r*), where *r* is the correlation coefficient. Heatmap shows the negative log scale, higher value indicates more spectrally similar condition pairs. (b) Endmember extraction using N-FINDR. 32 images of cells are pooled together and applied to an *n* = 3 N-FINDR feature extraction. A mask of cell vs. no-cell area was used to eliminate background interference with the algorithm. Three endmember spectra are found, with the last being noise. (c) Two-color reconstruction of the two significant endmembers with non-negative least-squares fitting. Each endmember image has a dynamic range between 0 and 1. Scales were applied to increase visual contrast.

These observations provided a view of the overall effects of fixatives on signal magnitude and the spectral characteristics of the SRS signal. Two fixatives, PFA and GA, preserve the spectral signature of unfixed live cells better than other fixatives, while exerting opposite effects on overall signal strength.

### Two-Channel Imaging Identifies Fixative-Specific Alteration of Lipid and Protein Content

To better understand chemical differences induced by fixation, we isolated component spectra contributions through unmixing via the N-FINDR algorithm.^26,51^ The number of endmembers, i.e., the chemical species, is selected empirically based on the number of nonarbitrary components found. To determine how many endmembers to use, we first explored a simple principal component analysis (PCA) on the pixels of cells. The PCA signature (Figure S2) showed that 2 out of 3 principal components had meaningful features while contributing 90.6% of the explained variance. In addition, based on the prior of protein and lipid being major cell constituents, three components were used. The third component is to account for N-FINDR’s sensitivity on outliers, with the one extra component being noise.

We then implemented an *n* = 3 N-FINDR in a reduced pixel subset from unfixed cells only, in order not to include any fixation artifacts in the spectral features. The extracted components have concordance with the Raman signatures of biomolecular species in the C–H stretching region: lipids (Figure 3b, endmember 1) and proteins (Figure 3b, endmember 2). This result is consistent with previous literature.^25,54,55^ However, the N-FINDR analysis is prone to noise and is unstable with respect to initialization. Therefore, the reconstructed spectra do not represent the true lipid and protein spectra and simply indicate distinct spectral components. The last endmember identifies the off-cell background pixels whose pixel values fluctuate from noise near the end of the power spectrum (see zoom-in view in Figure S3).

The chemical significance of the first two endmembers was further validated through two color abundance maps using nonnegative least-squares fitting (NNLS) (Figure 3c, Figure S5, Figure S6). Here, the two endmembers effectively represent protein and lipid content in cells in a two-color recolored image. This full spectral decomposition justifies a lower-dimensional representation of our hyperspectral data by focusing on protein and lipid effects, choosing the spectral bands at 2850 and 2920 cm^–1^ as the contributions with the least amount of cross-talk (See supplementary Figure S4). These bands offer high contrast for robust discrimination between lipid- and protein-rich regions and are well-established in the literature as markers for lipid^1,3,23,56^ and protein^23,28^ content.

To visualize the relationship between the extracted endmembers and individual Raman transitions, we calculated the similarity between each endmember-wavenumber pair. One challenge of comparing them is the choice of a uniform metric agnostic to absolute signal intensity and abundance. In order to achieve this, for each image sample (hyperspectral cube), we calculated the zero-mean normalized cross-correlation (ZNCC) between every pair of endmember-reconstructed image and original image taken at a certain wavenumber. By iterating all the images we have taken, we found that endmembers 1 and 2 are best-represented by images taken at approximately 2840 cm^–1^ and 2940 cm^–1^, respectively (Figure S7). This implies that a two-color image could capture the spatial features of endmember abundance.

We further investigated the effects of fixative on protein and lipid content, by constructing two-wavenumber proxies. Two-color composite images are generated from the hyperstacks by isolating protein and lipid signals from the 2850 cm^–1^ and 2920 cm^–1^ channels. To achieve clear separation, the pure protein signal from the 2920 cm^–1^ image is extracted by subtracting a rescaled 2850 cm^–1^ image, ensuring both images share the same intensity range. Six fixation conditions are visualized on a consistent color scale to highlight the linear dependence between signal intensity and component concentration of SRS (Figure 4a–f). For quantification, the raw images at each wavenumber were used for accuracy.

**Figure 4:**
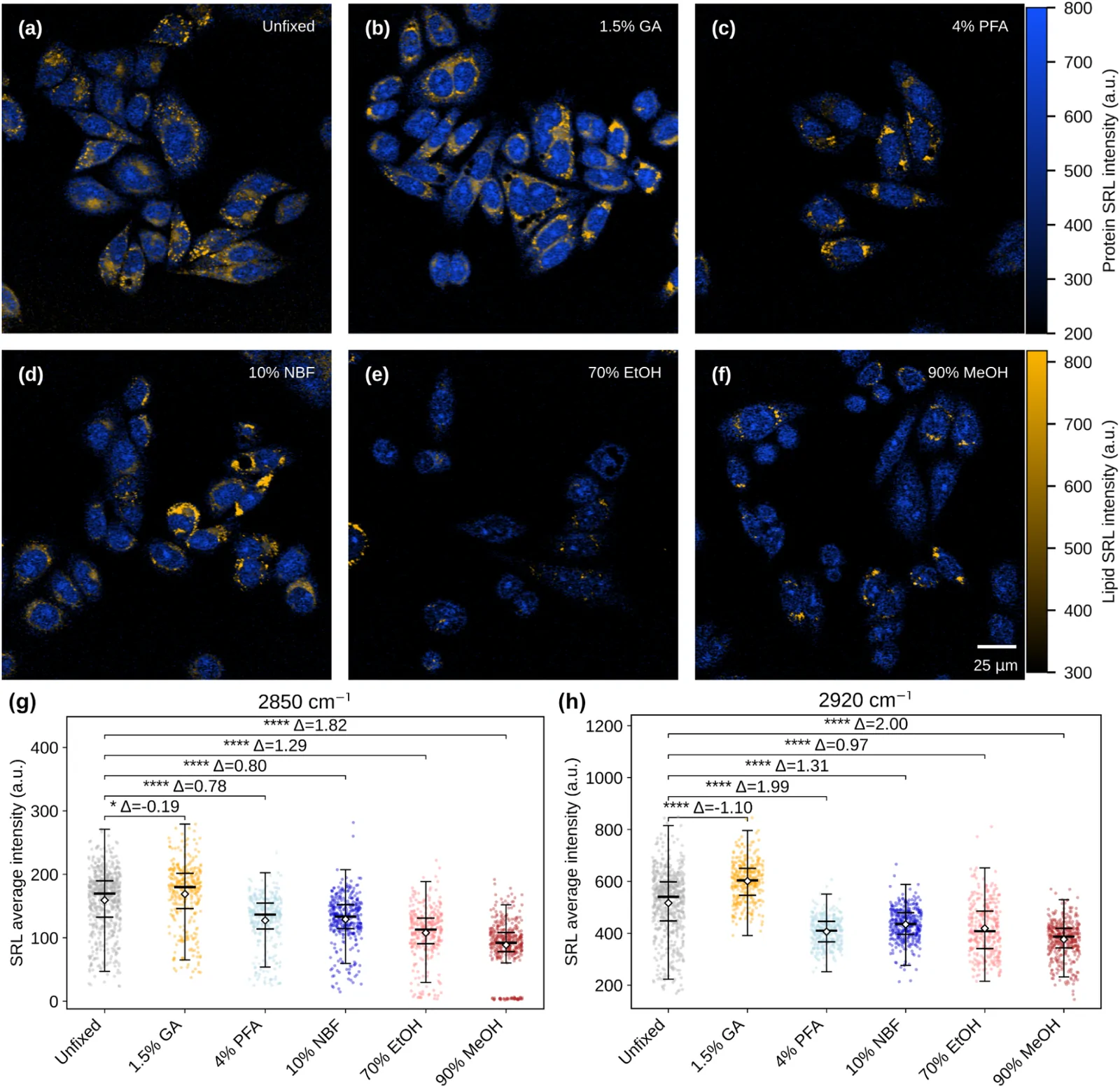
Two-color SRS images reveal fixation induced protein and lipid concentration changes on HeLa cells. (a–f) Representative two-color composite images of the lipid (2850 cm^–1^) and protein (2920 cm^–1^ subtracted with normalized 2850 cm^–1^) channels in six different sample preparation conditions, respectively: live (unfixed), glutaraldehyde, paraformaldehyde, neutral-buffered formalin, ethanol, and methanol. (g, h) Annotated strip plots of two characteristic Raman wavenumber signal distributions (2850 and 2920 cm^–1^) of all the cells in a specific sample preparation category. Each replicate experiment involved *n* > 376 cells. For each condition, diamond represents the mean, thick line represents the median, the two adjacent lines represent 0.25 and 0.75 quantiles, and the whiskers represent 1.5× the interquartile range. *, **, ***, and **** indicate *p* < 0.05, 0.01, 0.001, and 0.0001 (Tukey’s HSD), respectively. Δ noted as Glass’s delta.

#### Cross-Linking Fixatives Preserve Protein Concentration Closest to Live Cells

Live cells (Figure 4a) show bright and uniform protein signals (blue) throughout the cell. Glutaraldehyde-fixed cells similarly show intense and uniform protein signal (blue), with a signal distribution on average more intense than the live counterpart. Both PFA and NBF, with slightly fainter blue colors, show a small decrease in average protein signal and a less uniform protein arrangement, with significant structural reorganization characterized by the appearance of different bright internal nuclear structures (Figure S8c,d). Alcohol fixation (Figure 4e–f) yields the most variation compared to live cells, with an overall reduction in protein signal and a more localized signal intensity in the nuclear area, where fibrillar aggregates are clearly visible (Figure S8e,f). The images agree with analyses (Glass’s delta) of both 2850 cm^–1^ and 2920 cm^–1^ SRL average intensity at single-cell level (Figure 4g, h, S9), and the trend is consistent across replicates (Figure S9).

#### Alcohol-Based Fixatives Cause Consistent Lipid Loss

Live cells show bright and small lipid aggregations (gold) distributed mainly at the edges of the cell. Glutaraldehyde-fixed cells show an intense lipid signal (gold), with signal distribution on average more intense than live case. Among other fixation conditions, lipids in PFA-fixed cells show less significant change with respect to unfixed cells, making it a more preferable method at preserving lipid content. For NBF-fixed cells, the overall lipid loss extent is comparable to those fixed with PFA as validated through analysis on the 2850 cm^–1^ channel (Figure 4g), with the slightly fainter lipid signal possibly because of washout coming from its methanol content (up to 2%). Alcohol fixatives most likely cause lipid solubilization and washing away,^44,45^ with ethanol exhibiting some lipid loss and aggregations into larger structures with respect to unfixed. In the case of methanol fixation, lipids are not visible and are almost completely washed out. This observation is further supported by the ZNCC calculations (Figure S10).

#### Protein and Lipid Concentrations Shift across Major Cell Populations

To show that the effects of overall protein and lipid concentration change are not affected by outliers, we investigated the population-level metrics for the quantifications. First, we treated each cell as a data point and calculated the coefficient of variation (CV) for both 2920 cm^–1^ and 2850 cm^–1^ SRS signal, grouped by fixatives (Figure S11). Instead of seeing a consistent elevation of CV, the 2850 cm^–1^ CV remained stable for nonalcohol fixatives, while the CV for 2920 cm^–1^ decreased compared to the unfixed cell population. Along with kernel density estimation (KDE) plots (Figure S12) directly depicting the distribution, we can conclude that the shift effect is relatively uniform across cells for both protein and lipid concentrations.

#### Glutaraldehyde Fixation Exhibits Minimal Impact on Cell Size

The average unfixed cell size, measured by area, is 488 μm^2^ (Figure 5a, Table S4), which agrees with the average cross section of HeLa cells grown in nonhuman serum (370 ± 100 μm^2^).^57^ Glutaraldehyde fixation results in a slight decrease of cell size. Both PFA and NBF exhibit slight size increase and swelling, possibly due to rehydration in PBS, with PFA showing a slightly greater perturbation. Considerable shrinking can be observed in methanol fixed cells, while ethanol-fixed cells shrink only slightly. To support the statistical significance claims, we evaluated size variation across different sample replicates and imaging days for unfixed cells only (Figure S9, S13).

**Figure 5:**
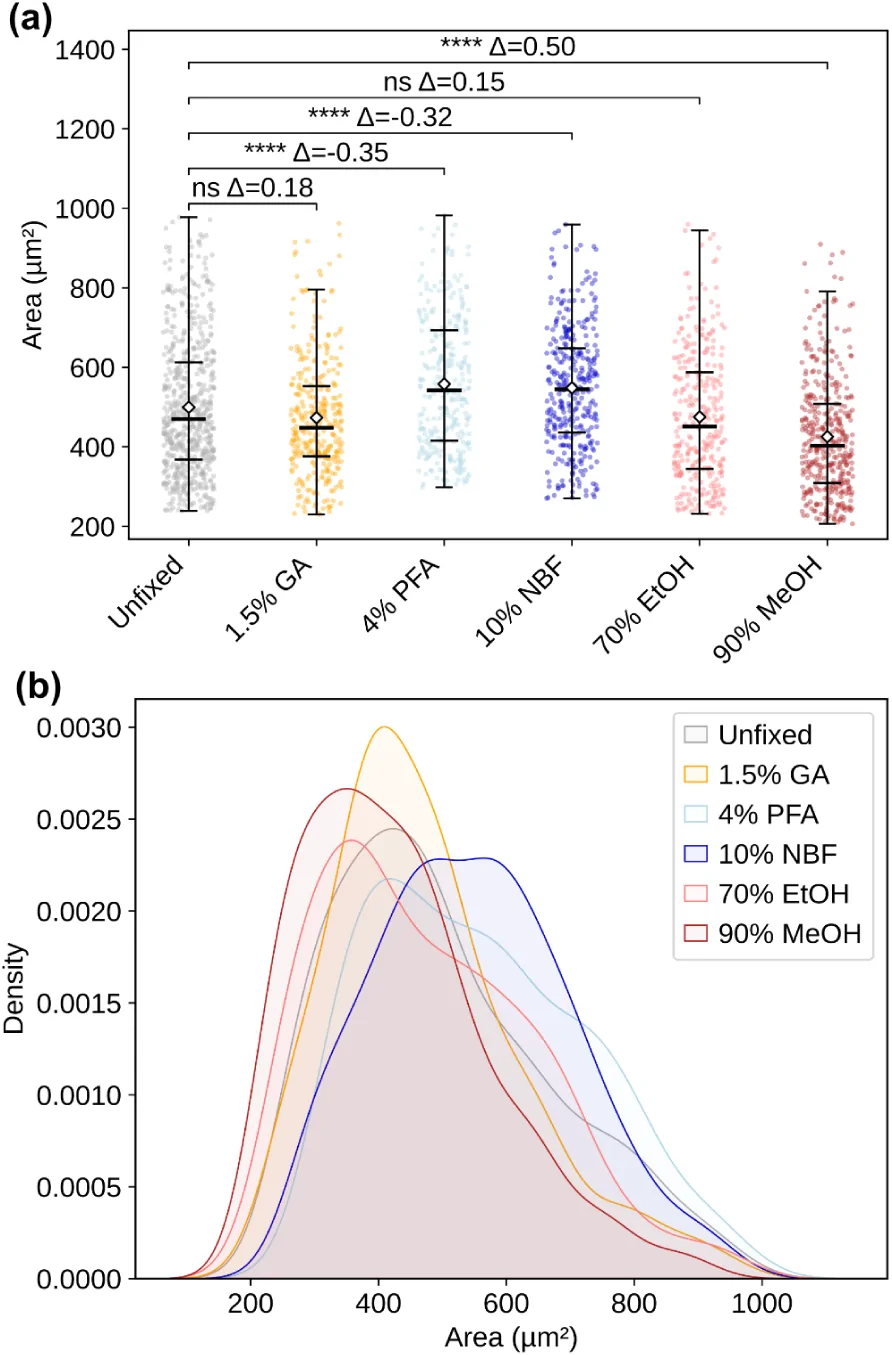
Fixation-induced cell size distribution change. (a) Annotated strip plots of cell size grouped by fixation conditions. For each condition, diamond represents the mean, the thick line represents the median, the two adjacent lines represent 0.25 and 0.75 quantiles, and the whiskers represent 1.5× the interquartile range. *, **, ***, and **** indicate *p* < 0.05, 0.01, 0.001, and 0.0001 (Tukey’s HSD), respectively. Δ noted as Glass’s delta. (b) Kernel density estimation plots of the cell size distribution, grouped by fixation conditions. Gaussian kernels with bandwidth estimated by Scott’s rule were used.

Although both GA and EtOH exhibited no statistical significance, we observed that the size distribution of EtOH-fixed cells was wider and more bimodal (Figure 5b), different from those in GA-fixed and untreated cells. Given the statements above, we can conclude that glutaraldehyde has an overall minimal effect on the cell size distribution.

#### Formaldehyde-Based Fixatives Retain the Spatial Distribution of Lipids within Cells

While the observations above focus on cell-to-cell heterogeneity, we also investigated the effect of fixatives on intracellular biomolecule distribution. As an example, we chose the spatial distribution of lipids for demonstration, using SRS images of the 2850 cm^–1^ band as a proxy of the lipid concentration map.

Similar to the quantification of the SRS signal over cell populations, the coefficient of variation (CV) could also be used to quantify the overall extent of spatial dispersion or clustering of the signal. By extracting a per-cell CV score, we were able to compare the CV distribution between fixation conditions (Figure 6a). Notably, cross-linking fixatives showed no significant alteration of lipid dispersion, whereas alcohol-based fixatives showed an increase. This effect is conjunct with the lipid loss, which is logical given how the CV is calculated.

**Figure 6:**
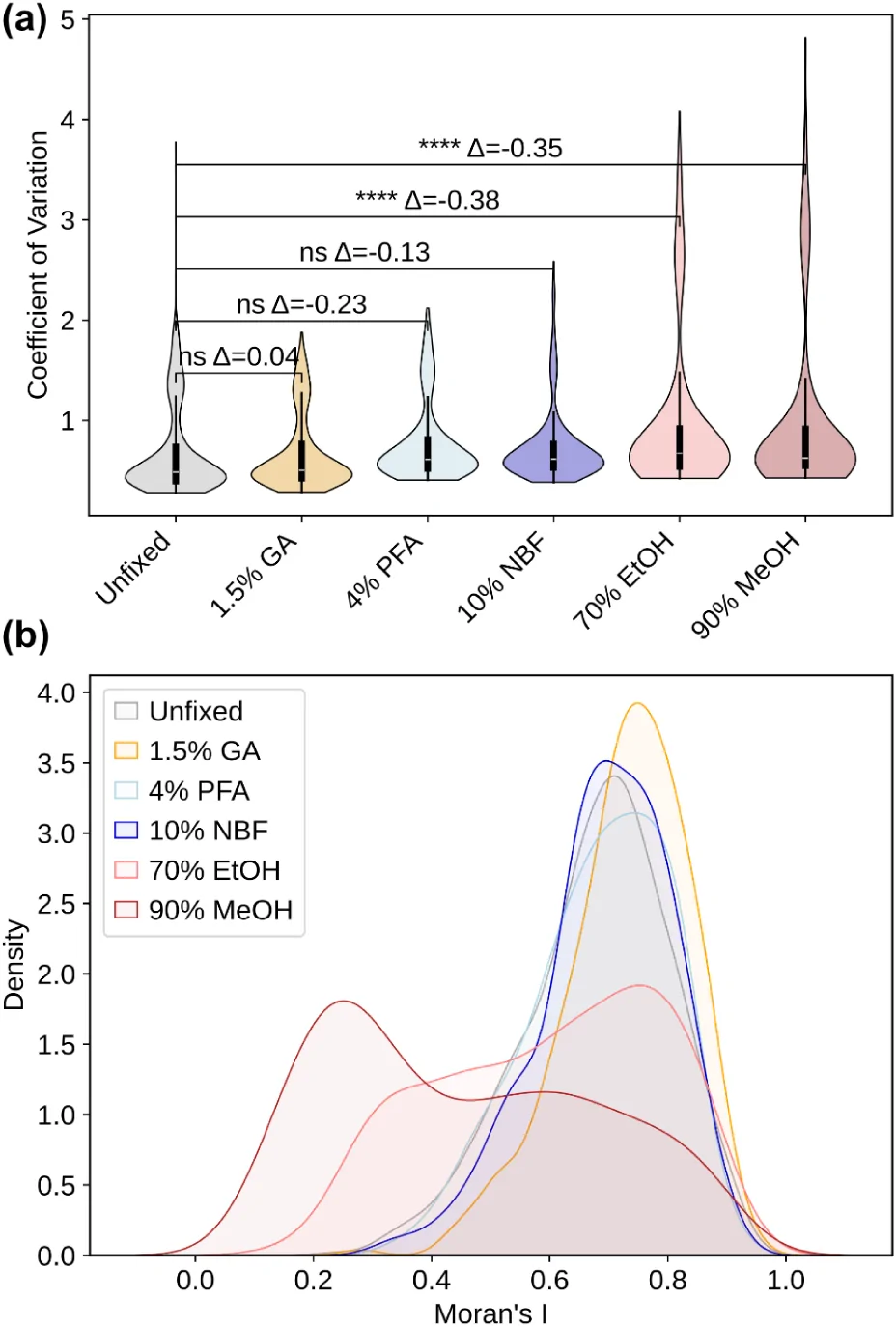
Fixation-induced lipid signal clustering level characterization. (a) Violin plots of coefficient of variation (CV) of single-cell 2850 cm^–1^ (mainly lipid) signal. A per-cell CV metric was calculated, and aggregated for plotting. A cell with spatially clustered signal will have a higher CV. For each condition, the white dot represents the median, the thick bar represents the middle 0.5 quantile, and the whiskers represent 1.5× the interquartile range. *, **, ***, and **** indicate *p* < 0.05, 0.01, 0.001, and 0.0001 (Tukey’s HSD), respectively. Δ noted as Glass’s delta. (b) Kernel density estimation plots of the cell lipid signal spatial autocorrelation (Moran’s I) distribution. Higher Moran’s I indicates a more clustered signal. Gaussian kernels with bandwidth estimated by Scott’s rule were used.

Although CV is a computationally efficient metric, it does not take spatial context into calculation. Therefore, we used Moran’s I (spatial autocorrelation) to compare the fixatives from another aspect. From the distribution plots (Figure 6b) we observed an increase in clustering extent among cells that were GA fixed, while PFA and NBF preserved almost identical distribution compared to nonfixed cells. This claim is supported by statistical tests (Figure S14). Thus, in terms of lipid spatial distribution, the two formaldehyde-based fixatives are considered better than GA.

## Discussion

Here, we used stimulated Raman scattering to investigate the effects of biological fixation on cultured cells to aid in choosing the fixation technique to use in vibrational microscopy. We expand on previous studies characterizing biological fixation techniques in bacteria^14^ and various cell strains^13,17,18^ that use spontaneous Raman spectroscopy in the fingerprint region and extend the analysis to the C–H stretching region through hyperspectral SRS microscopy. We provide a more comprehensive characterization of fixation-induced changes through high-resolution, quantitative measurements of chemical and morphological features, combining analyses of Raman spectra, spatial distribution of biomolecules, and cell-population-level statistics.

Using a cell model, we characterized changes in the size and chemical composition across different sample preparations. We further identified consistent patterns of fixation mechanisms by analyzing a large number of cells and performing imaging over a range of days to overcome batch effects. Our study provides guidelines for future stimulated Raman scattering microscopy experiments that require fixation or design in tandem with other multimodal techniques.

First, our observations confirm the considerable disruptive effects of alcohol fixation, as a significant portion of cellular lipid signals is lost. Images taken after alcoholic fixation typically require denoising algorithms to recover spectral features, making them suboptimal methods for SRS microscopy.^58,59^ In addition, significant structural reorganization was observed (see Figure 4e,f, S8e,f), likely due to protein precipitation that leads to a proportional increase (up to 40%, reported in the literature) in concentrations of nucleolar proteins and RNA. Possible reasons include cell shrinkage and the formation of intracellular granules with localized increases in the cytoplasmic lipid signal.^8^ Moreover, protein denaturation leads to unfolding, exposing or reorienting CH_3_ groups, resulting in aggregation and structural changes that reduce the average protein signal.^33,46^ These results align with the understanding that cross-linking fixatives best maintain protein conditions similar to live cells.^9,17,33^ When coupling an SRS microscopy experiment with histological applications or other downstream analyses requiring alcohol fixation, e.g., spatial transcriptomics, significant morphological changes and lipid loss must be taken into account.

Glutaraldehyde shows promise as a candidate fixation method for SRS due to the significant signal increase both in lipid and protein, in agreement with previous works,^27^ at the same time showing minimal spectral shape distortion (Figure 2g, 3a, 4b, g–h, 5a). Among all the fixatives, GA is one of the two conditions that did not significantly change cell area on microscope images. This makes it potentially preferable in morphology applications of SRS given that ethanol, while preserving size, washes away lipid signal from cells. GA’s feature of signal increase coupled with no significant cell area change could suggest a decrease in cell volume, i.e., flattening, resulting in higher cell density,^53^ which was not quantified here given the single *z*-slice images acquired. Further investigation performing 3D imaging could validate this hypothesis, ensuring that the observed effects are not spurious signals introduced by GA binding but are only of morphological nature. It is worth noting that, compared to the other two formaldehyde-based fixatives, GA increases the spatial autocorrelation (Moran’s I) of the lipid signal by a significant amount (Figure 6b, Figure S14), implying a stronger cross-linking capability of biomolecules such as lipoproteins. When treating the spatial distribution of lipids as an observable, GA needs to be used with caution as a fixative.

In agreement with previous work, we observed that formaldehyde-based fixatives may reduce protein concentrations by 5% to 18%, with potential lipid alterations or loss.^8,13,17^ Besides, a slight size increase was measured, due to swelling following rehydration in PBS (Figure 5a), however, despite the drawback above, they did not exhibit significant perturbation of the internal spectral (Figure 3a) or spatial (Figure 6b) structure of the cell. Between the two fixatives, PFA performs slightly better on maintaining spectral features, while NBF retains more protein signal (Figure 4h). In order to combine the benefits from both GA and PFA, it is also possible to mix the two fixatives,^35^ though the actual recipe might vary depending on the biological sample.

Our experiment quantified the effects of fixation on SRS microscopy on a HeLa cell model, informing other cell lines’ possible trends. Meanwhile, there are certain caveats of the study worth noting. First, the study is conducted on a population of cells, and the *in situ* effect of fixation effect of fixation was not characterized. In order to achieve a more rigorous characterization of fixation effects, imaging the same cell before and after fixation on SRS needs to be performed. This would pose significant challenges on experimental setup but would provide more accurate paired data. Second, certain morphological biases could be introduced during the experiment. For example, each FoV undergoes manual focusing, which could deviate between different microscope operators; while doing microscopy, cells with visible membrane disruption and blebbing could be selectively excluded, shifting the quantification results. A more robust way to eliminate the biases is to use the same slides for multiple sample preparation and also scan the sample along the *z*-axis. This requires careful experimental design given that live cells have a short life in imaging solutions like PBS, and *z*-scanning further limits the data acquisition throughput. Third, while the C–H stretching region SRS imaging of HeLa cells is generalizable to a lot of applications, our results might not hold depending on the sample and detection range, such as lipid spatial distribution in adipose tissue, and the abundance of certain sharp fingerprint signatures.

For a more generalized characterization, the current study could be expanded in scope by testing various human cell models with a more diverse morphology, to further explore the extent of the effects, e.g., the high lipid content of adipocytes makes them particularly susceptible to lipid washout under alcohol fixation conditions. Moreover, this work can suggest possible effects of tissue fixation for SRS imaging, not including morphological factors like extracellular matrix/collagen, overall morphology in 3D, and depth-scattering effects coupled with exogenous clearing agents, e.g., paraffin or xylene, used to reduce scattering and allow higher penetration depth for imaging. For these reasons, choosing Raman-compatible clearing methods ensures optimal volumetric chemical imaging with a 10-fold increase in penetration depth without distorting the SRS signal.^60^ Here, we characterized cellular behavior focusing on proteins and lipids in the C–H stretching region, providing a basis for identifying potential trends when exploring other spectral regions, e.g., fingerprint region, or when using exogenous Raman labels to access the silent region, to study metabolites, or biomolecules.^61–63^ The selected concentrations, times, and temperatures are based on a modal sense of the wide variety of methods used in the literature. Any variation in these parameters may modulate the fixative-induced effects on SRS, with the patterns described here serving as a reference for interpreting the outcomes.

Despite the limitations, our findings provide essential insights into the effects of chemical fixation on cellular lipid and protein profiles within the C–H stretching region of the Raman spectrum. While previous studies have demonstrated qualitative effects of fixation on the stimulated Raman signal, we leveraged the power of label-free SRS microscopy to quantitatively characterize fixation-induced spatio-molecular changes, also providing a more authentic comparison against real unfixed live cells. This study represents a pivotal step in advancing SRS as a prime method for biomedical analysis and characterization, ensuring that cells or tissues are observed under the most pristine conditions. We envision the broader application of our findings to refine fixation protocols, enabling more accurate and reproducible Raman-based studies that enhance our understanding of cellular and tissue dynamics in health and diseases.

## Supplementary Material



## Notes

Additional tables and figures, including summaries of cell counts, 2920 cm^–1^ signal, 2850 cm^–1^ signal, and cell size distributions (Tables S1–S4, Figures S11–S13); fixative solution spectra (Figure S1); principal component analysis and N-FINDR endmember spectral extractions (Figures S2–S4, S7); two-color endmember recomposition images based on non-negative least-squares fitting (Figures S5, S6); zoomed-in view of 2-color images of individual cells (Figure S8); day-to-day reproducibility analysis via Glass’s delta (Figure S9); ZNCC lipid loss validation (Figure S10); spatial autocorrelation analysis (Figure S14) (PDF)
