# Supplementary material for: Effects of Chemical Fixation on Stimulated Raman Scattering Microscopy
Source: Anal Chem. 2026 Sep 23;98(39):28666–77. doi: 10.1021/acs.analchem.6c01478 (PMC13647981; doi:10.1021/acs.analchem.6c01478)
Supplement: Supplementary file 1 [file ac6c01478_si_001.pdf]

Effects of chemical fixation on stimulated Raman scattering microscopy

Chiara Ceconello[1,2†], Wuji Han[3†], Bryce Manifold[1], Dario Polli[2,4] and Aaron Streets[1,3,5,6*].

1 Department of Bioengineering, University of California Berkeley, Berkeley, California, 94720, USA
2 Department of Physics, Politecnico di Milano, Piazza Leonardo da Vinci 32, Milano, 20133, Italy
3 Center for Computational Biology, University of California Berkeley, Berkeley, California, 94720, USA
4 CNR-Institute for Photonics and Nanotechnologies (IFN-CNR), Piazza Leonardo Da Vinci 32, Milano, 20133, Italy
5 Biophysics Graduate Group, University of California Berkeley, Berkeley, California, 94720, USA
6 Biohub, San Francisco, California, 94158, USA
† These authors contributed equally
* correspondence: astreets@berkeley.edu

Supporting Information

Table of contents:

Fig. S1: SRL Raman spectrum of the fixative solutions
Fig. S2: Principal Component Analysis of hyperspectral SRS image
Fig. S3: Zoom-in view of the third endmember
Fig. S4: Spectral difference of endmembers 1 and 2 extracted using N-FINDR
Fig. S5: Images of the cells based on non-negative least square reconstruction of endmembers
Fig. S6: Two-color composition of the reconstructed endmember images
Fig. S7: Best-match wavenumber per N-FINDR endmember
Fig. S8: Zoomed-in view of 2-color images of individual cells
Fig. S9: Glass's delta of fixation conditions relative to unfixed across imaging sessions
Fig. S10: ZNCC verifies the effect of lipid loss in methanol fixed cells
Fig. S11: Coefficient of variation of 2920 cm^{-1} and 2850 cm^{-1} SRS signal across cells
Fig. S12: Distributions of protein or lipid concentrations across cells
Fig. S13: Size distribution of unfixed cells measured on different days
Fig. S14: Annotated strip plots of spatial autocorrelations (Moran's I)

Table S1: Summary of cell counts and replicates per condition of fixatives
Table S2: Summary of 2920 cm^{-1} band average intensity distributions
Table S3: Summary of 2850 cm^{-1} band average intensity distributions
Table S4: Summary of cell size distributions

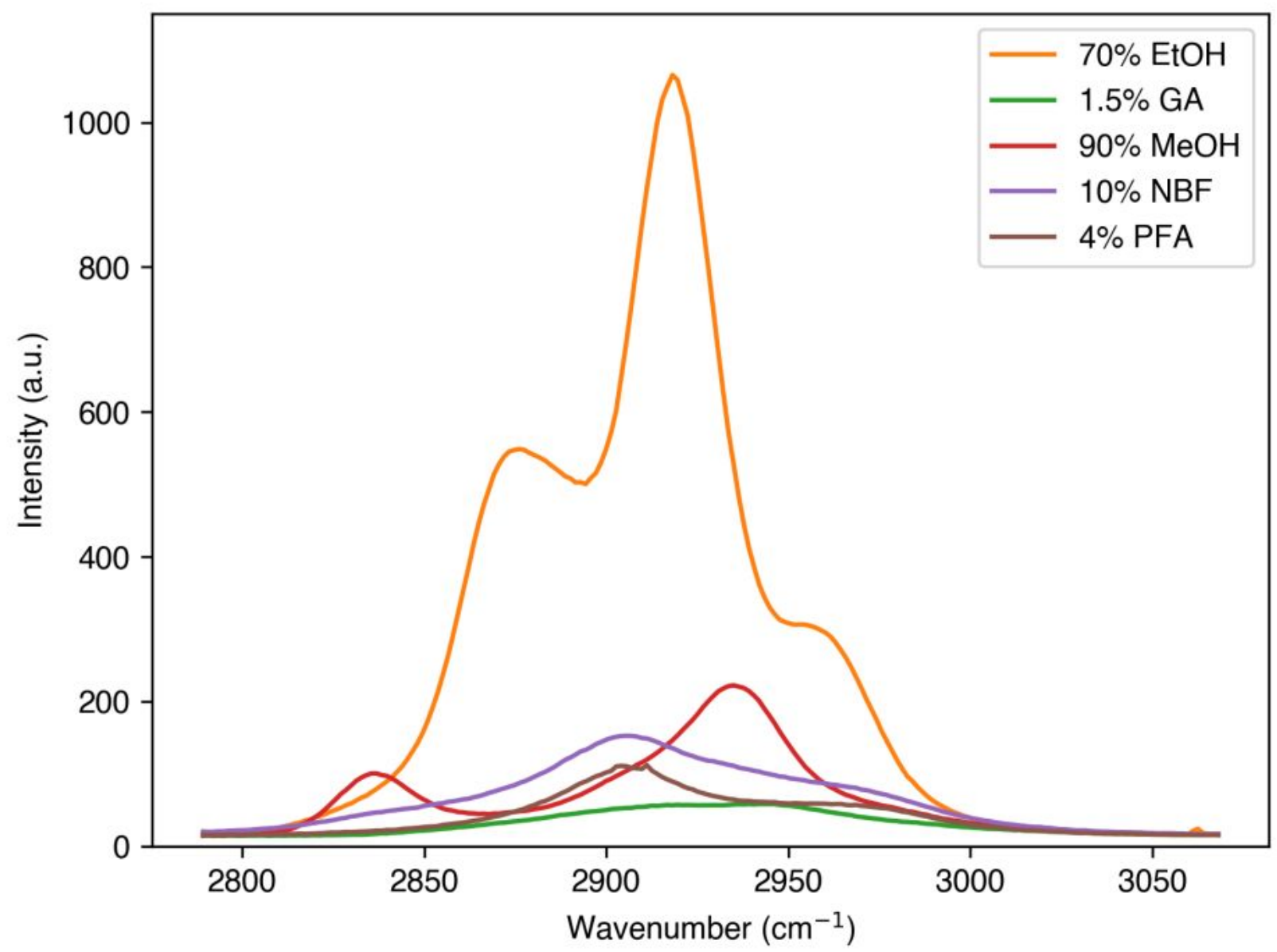

Fig. S1: SRL Raman spectrum of the fixative solutions. SRL Raman spectrum of the five fixatives in the concentrations employed in the study. See methods for fixative compositions. For this experiment, 50uL of fixative solution is deposited on a coverslip/microscope slide sandwiched configuration and a single-point SRL spectrum is acquired. Laser powers are set to 20/25 mW (Pump/Stokes), wavelengths are the same employed in the study (Pump at 799nm, Stokes at 1040nm) targeting the C-H stretching spectral region through spectral focusing. Water peak is outside of the accessible region through spectral focusing at the selected wavelengths.

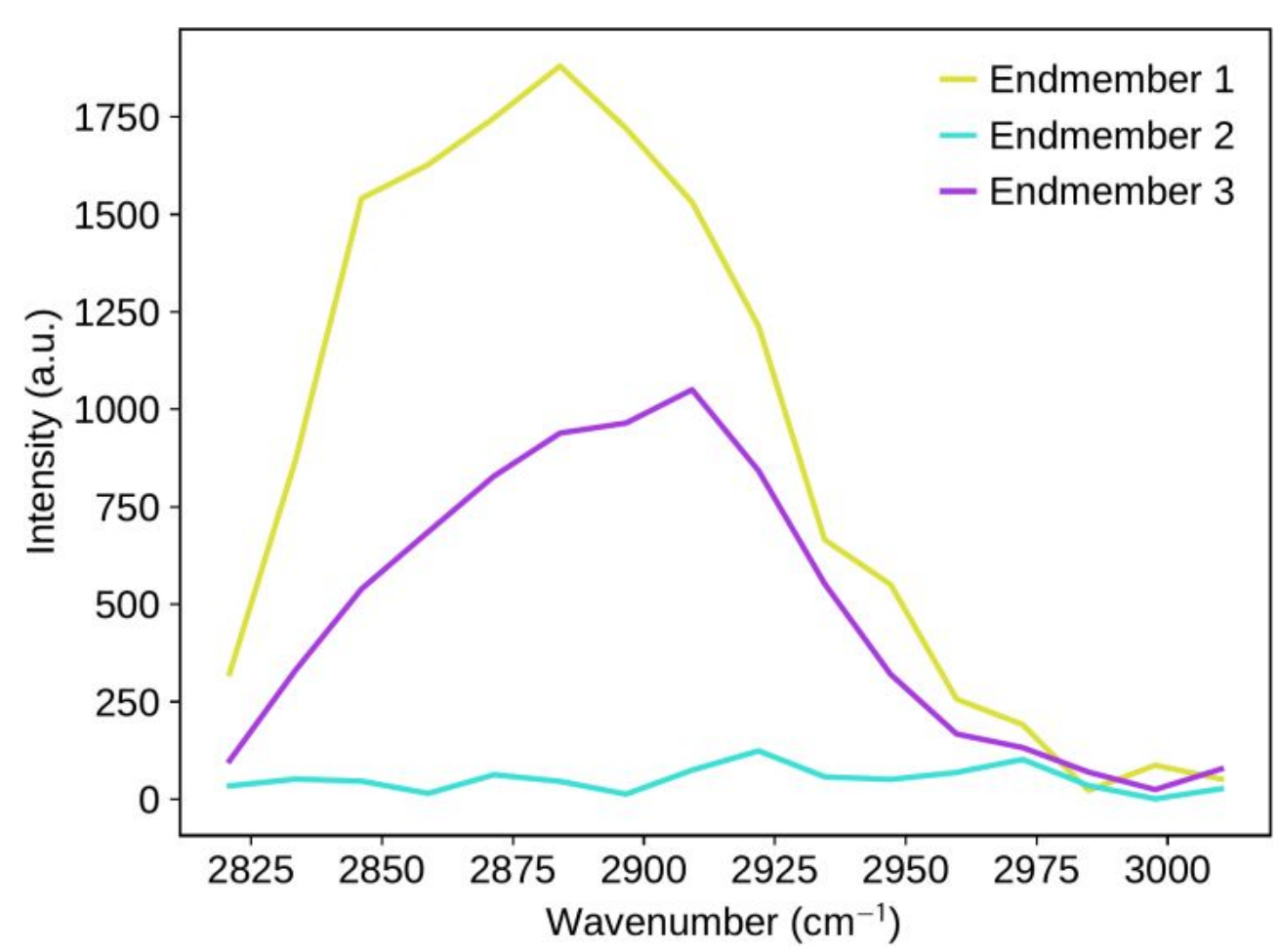

Fig. S2: Principal Component Analysis of hyperspectral SRS image. One example showing the spectral decomposition using PCA for cell-masked pixels (spectra).

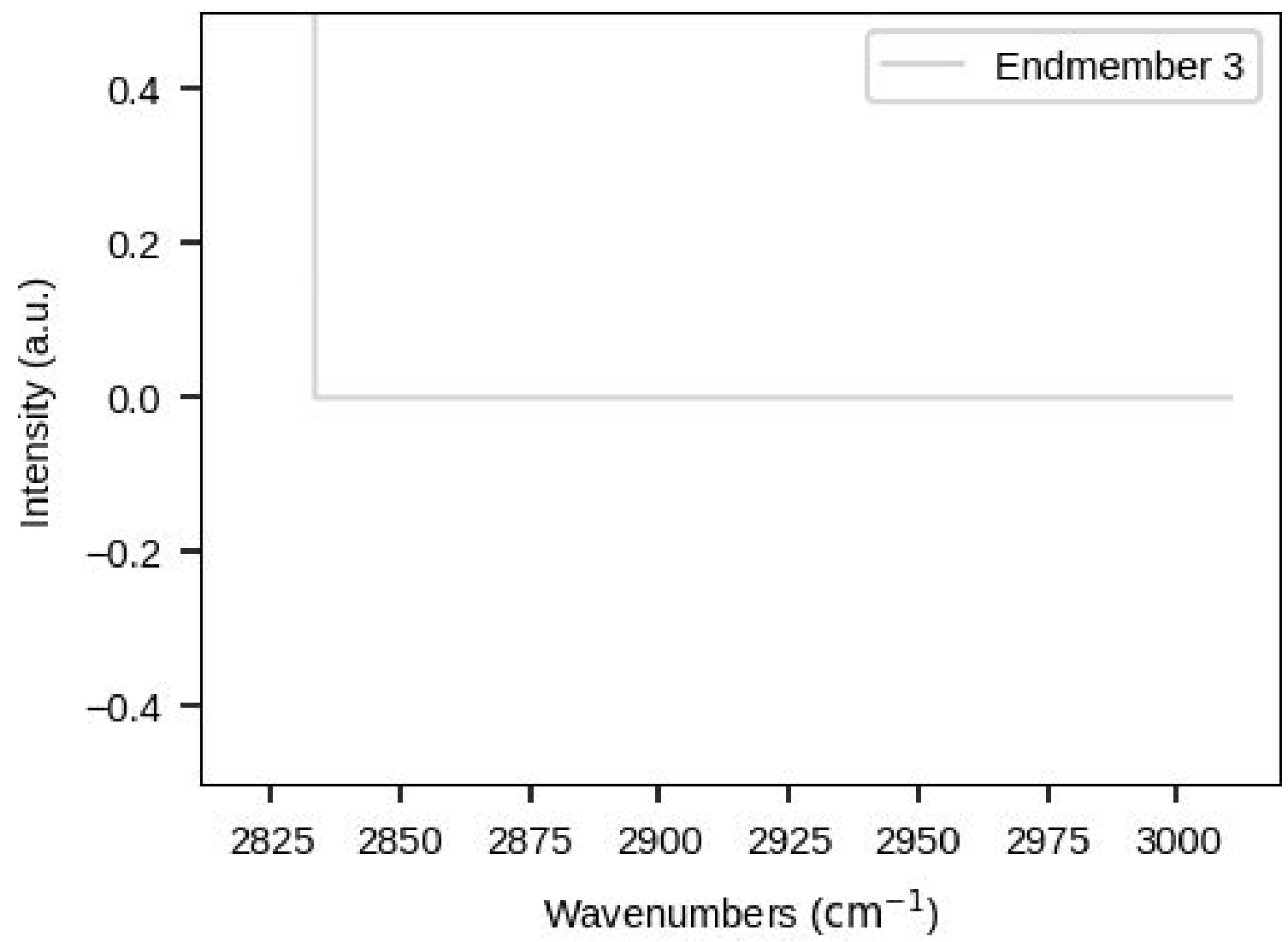

Fig. S3: Zoom-in view of the third endmember in Fig 3(b), contributing mostly noise from wavenumber near the edge of the power spectrum and having the least SNR.

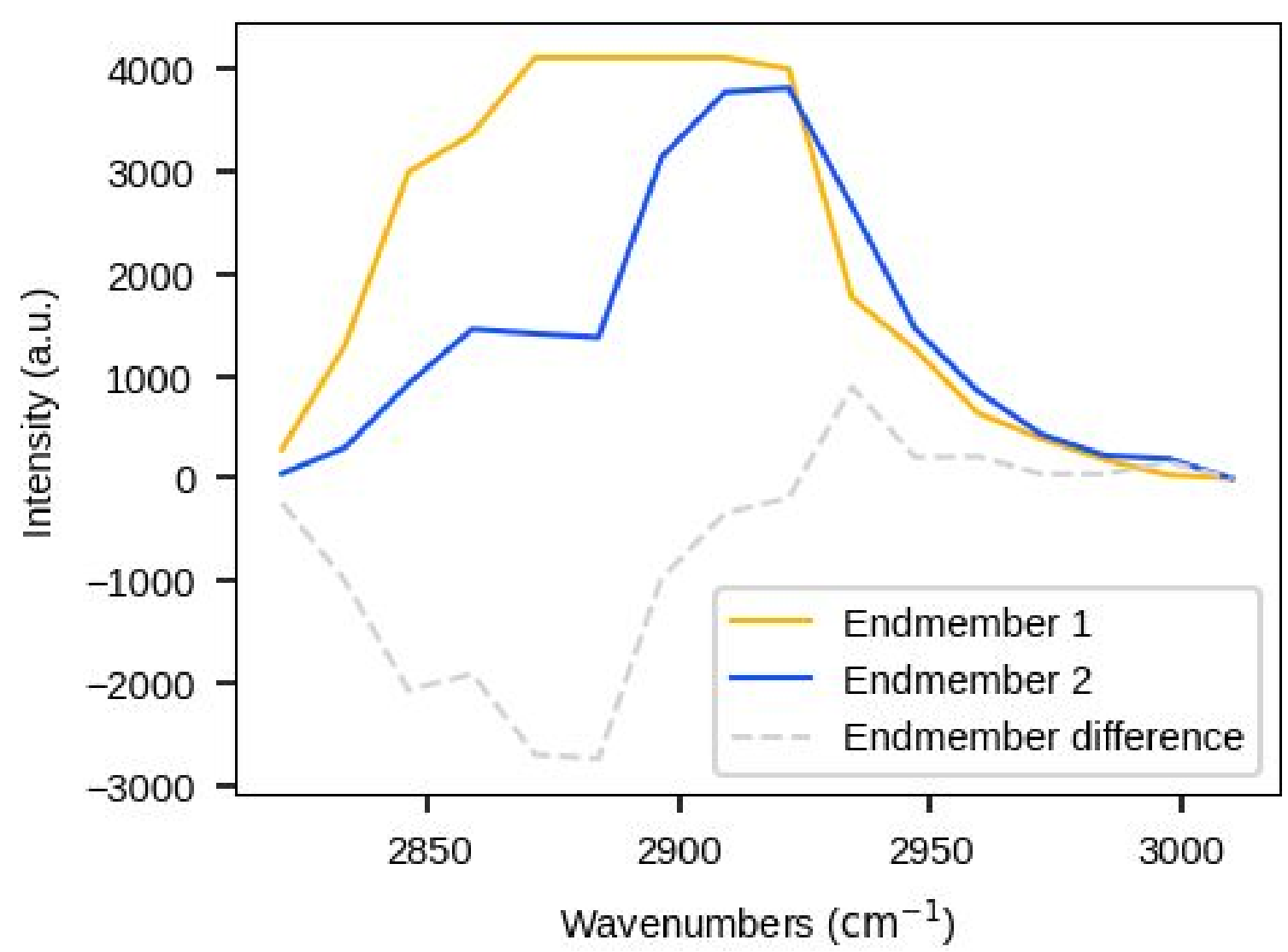

Fig. S4: Spectra of endmembers 1 and 2 extracted using N-FINDR, and their difference. The selection of 2850 cm^{-1} and 2920 cm^{-1} as representative bands for lipids and proteins, respectively, was guided by the difference spectrum, which highlights regions of maximal variation between the two components. These bands correspond to areas of high contrast and minimal spectral cross-talk, allowing robust discrimination between lipid- and protein-rich regions. Notably, 2850 cm^{-1} and 2920 cm^{-1} are also well-established markers for lipid and protein content in the literature.

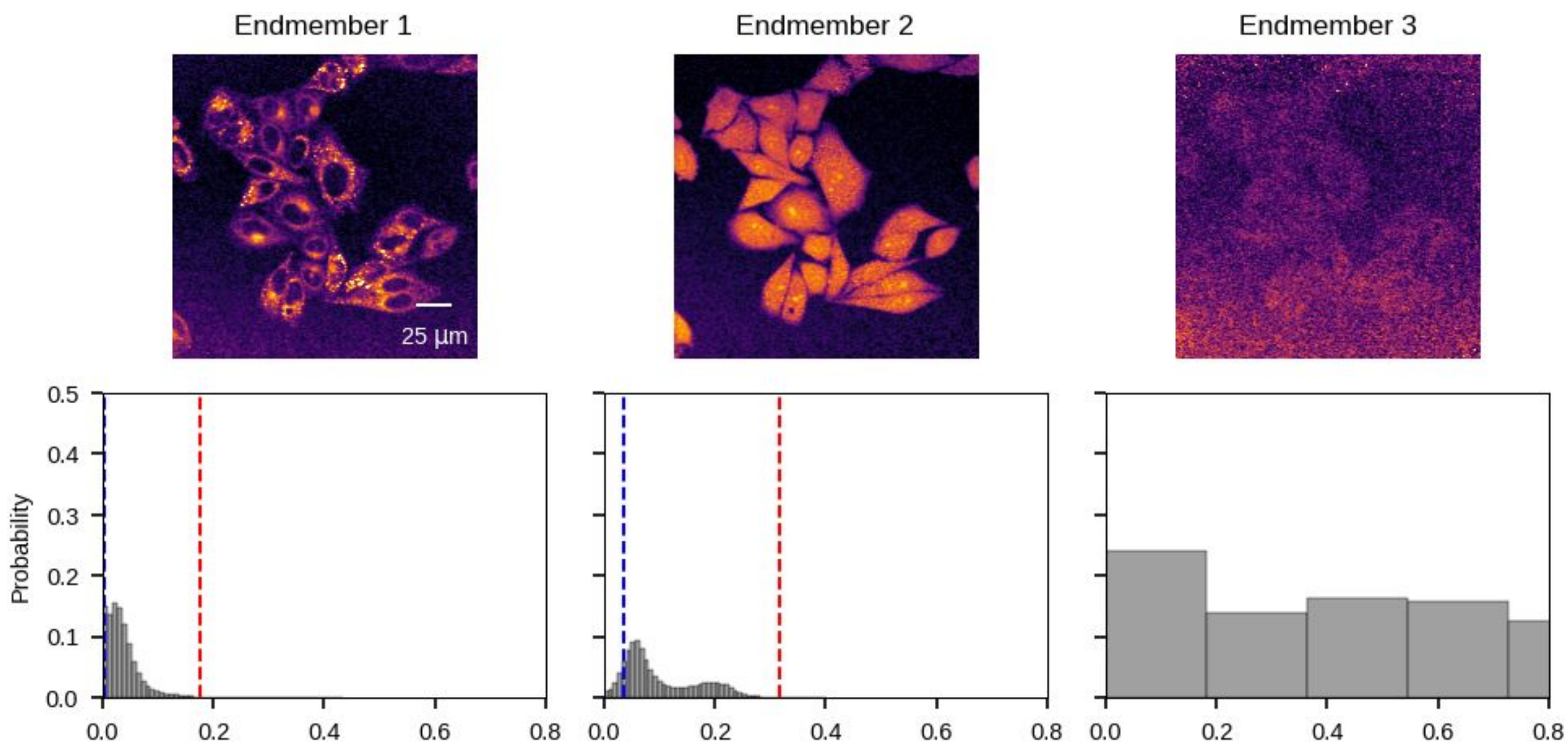

Fig. S5: Images of the cells based on non-negative least square reconstruction of endmembers. Bottom plots show the histograms of intensity distributions, as well as the dynamic range for two-color composition.

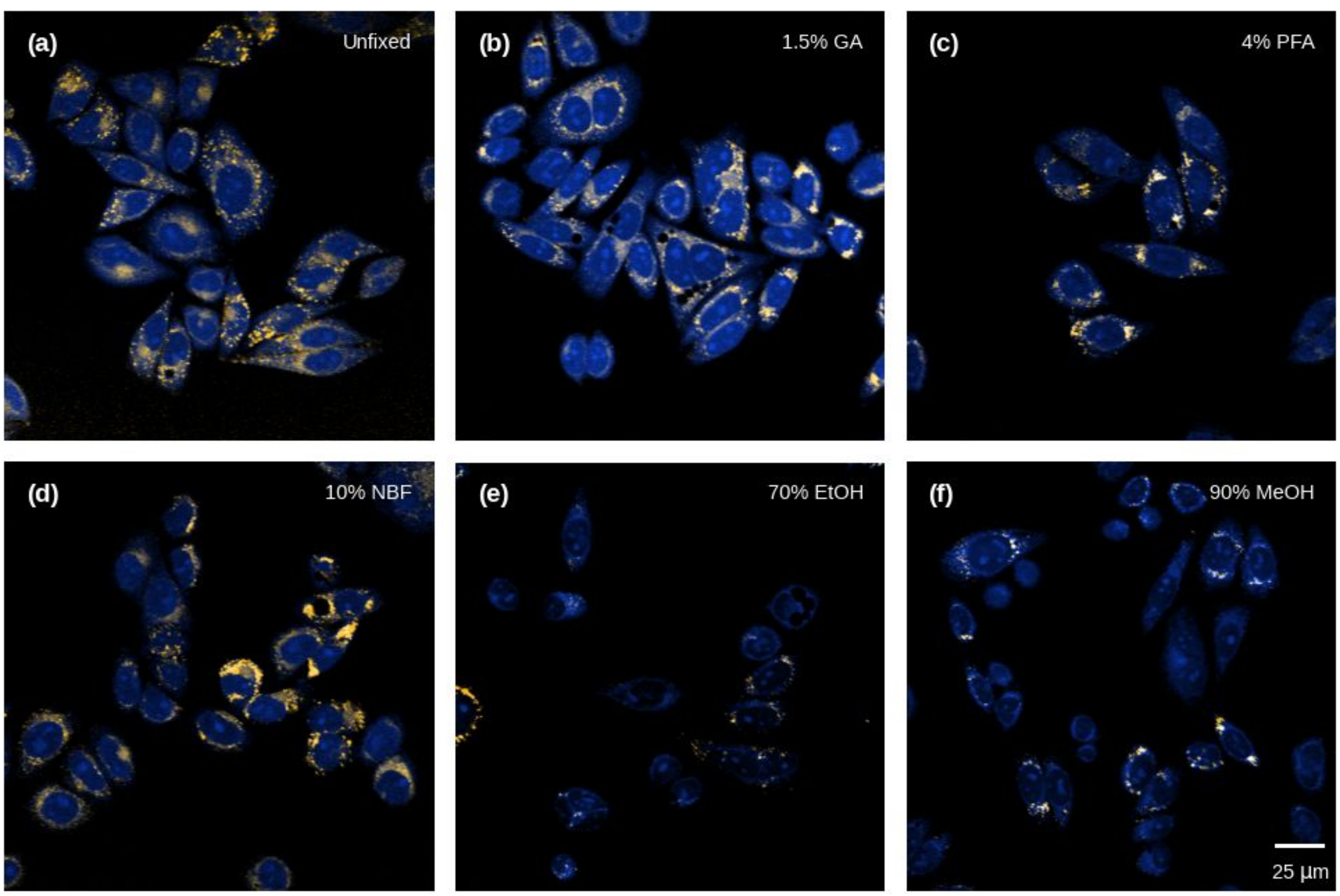

Fig. S6: Two-color composition of the reconstructed endmember images. The field of views were the same as shown in Fig. 4.

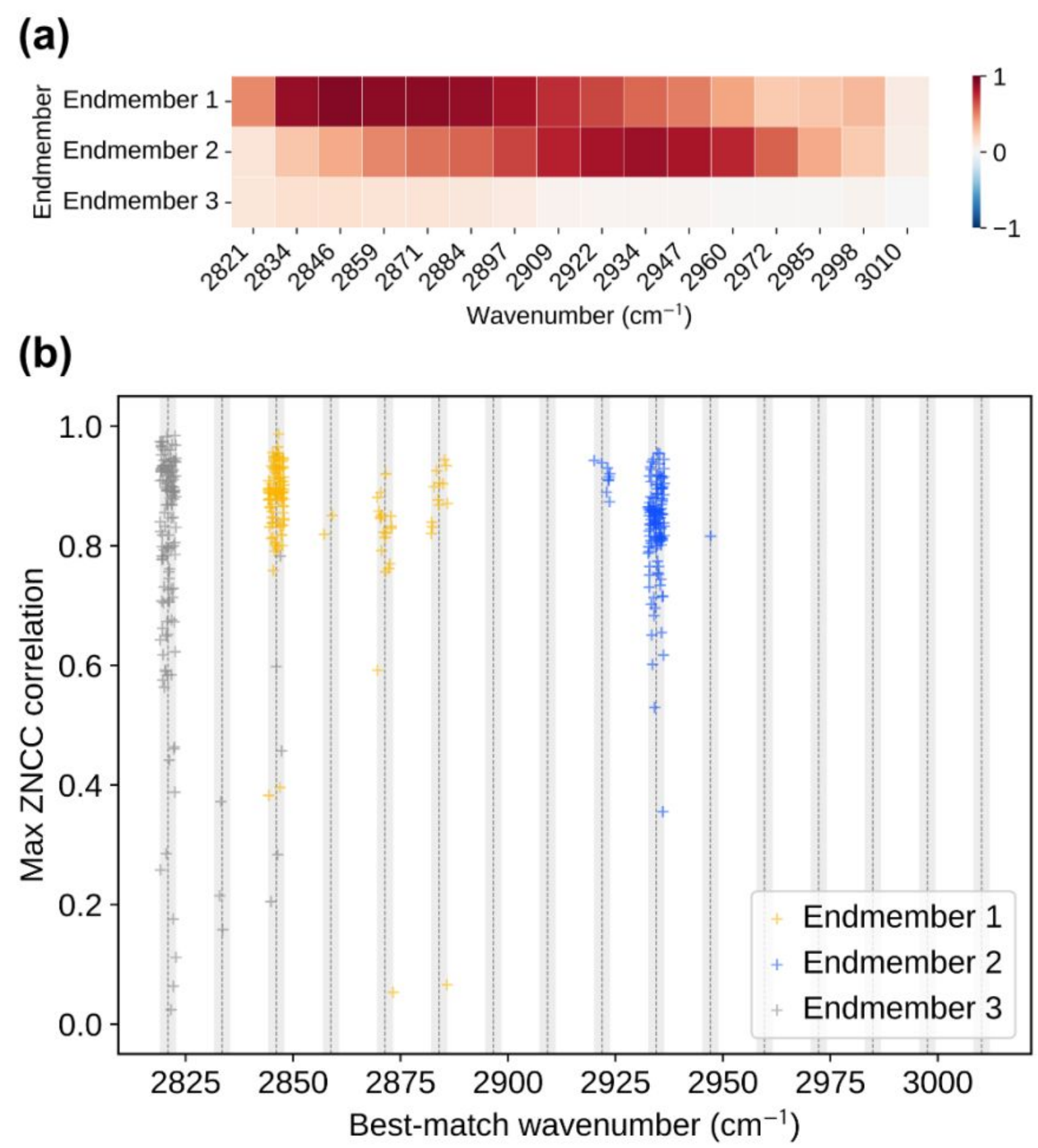

Fig. S7: Best-match wavenumber per N-FINDR endmember. Zero-mean normalized cross correlation (ZNCC) between abundance images reconstructed with endmember spectra and raw SRS images taken at a fixed wavenumber were calculated. (a) ZNCC ranges from -1 to +1, higher indicating the two images being more correlated. For endmember 1 and 2, 2846 cm^{-1} and 2934 cm^{-1} are the best-matching wavenumbers. (b) Aggregated view of best-match wavenumbers using all 136 cell images. 3×16×136 pairs were calculated, and the panel shows 3×136 best-matching wavenumbers. Crosses were slightly jittered to better depict abundance.

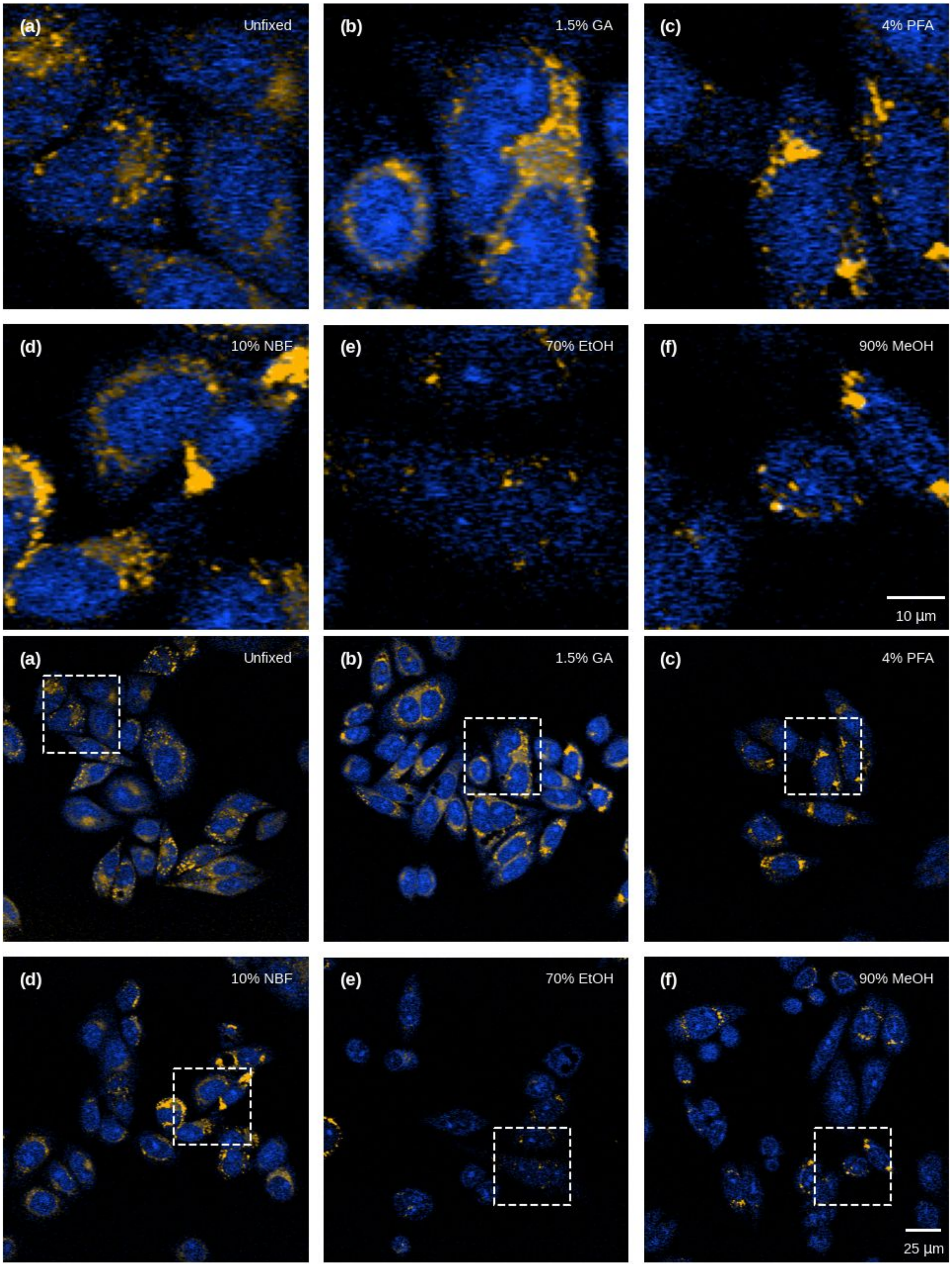

Fig. S8: Zoomed-in view of 2-color images of individual cells to highlight changes in nuclear aggregation and their corresponding region in Fig. 4.

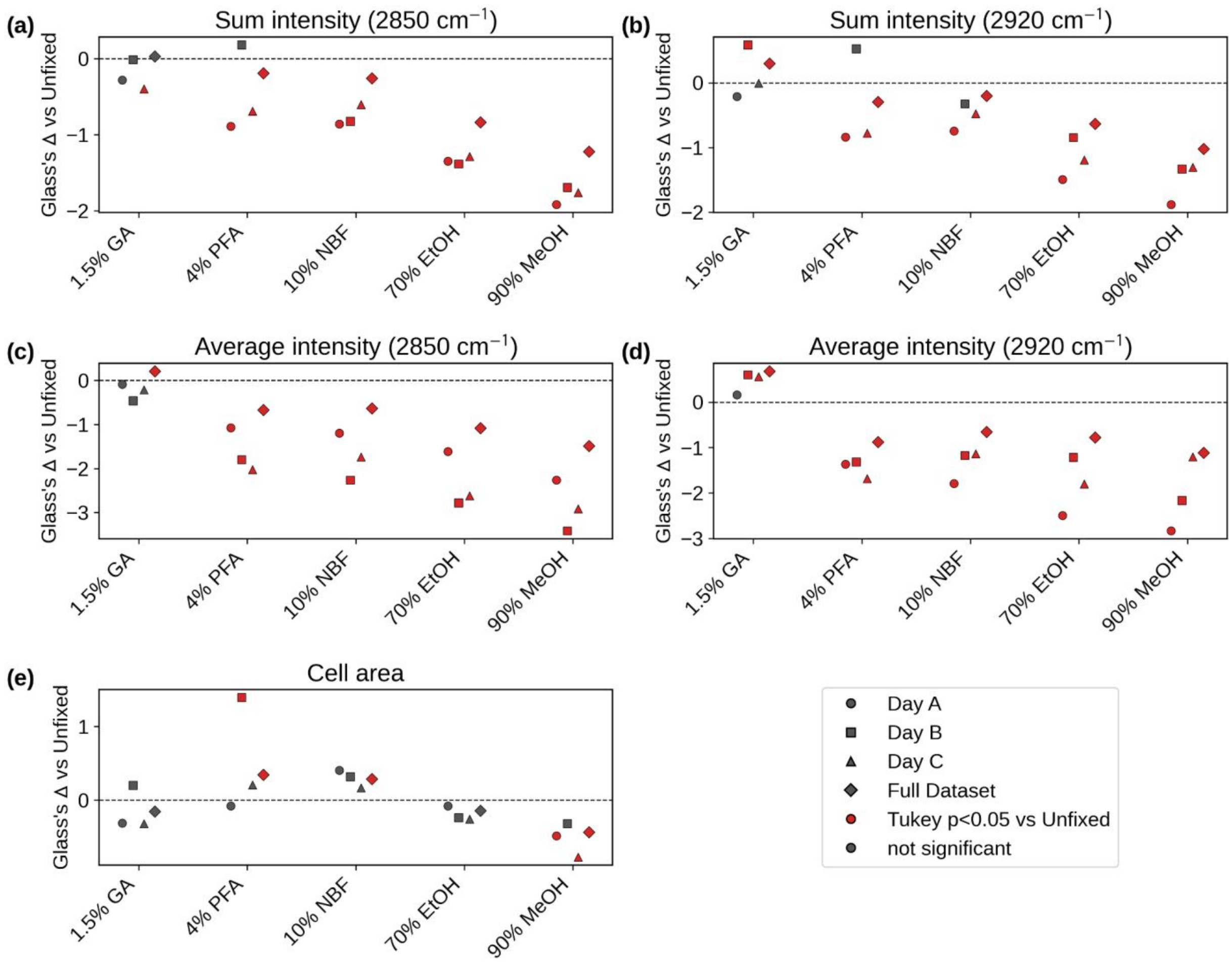

Fig. S9: Glass's delta of fixation condition measurements relative to the unfixed control across imaging sessions. (a,b) sum intensity, (c,d) average intensity, and (e) cell area. Markers show three imaging sessions on different dates (circle, square, triangle) in which all fixative condition measurements were performed, and the diamond marker indicates the full dataset in this study; red indicates Tukey HSD $p < 0.05$ vs. unfixed.

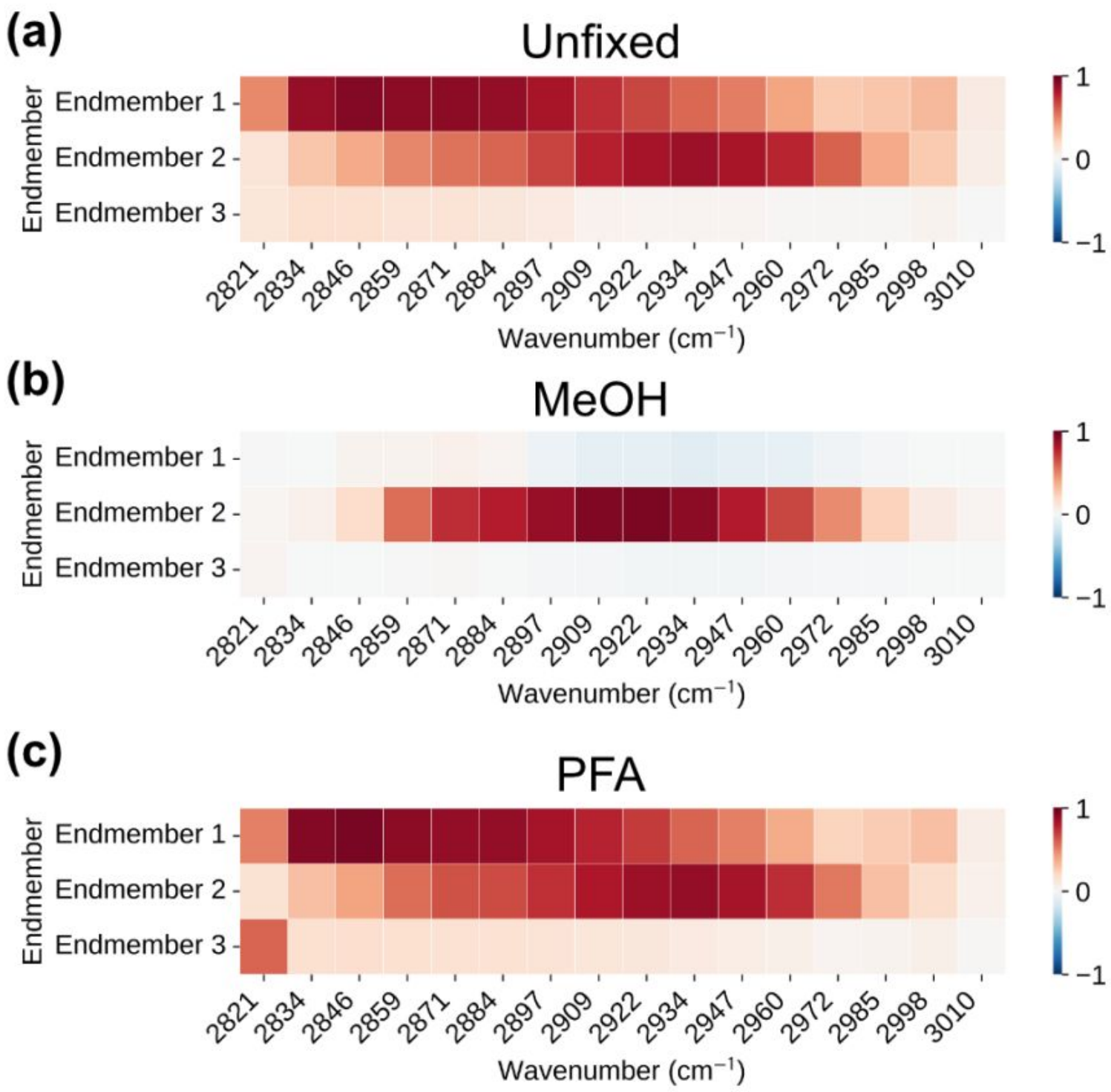

Fig. S10: ZNCC verifies the effect of lipid loss in methanol fixed cells. The correlation between 2846 cm^{-1} and endmember 1 drastically diminishes under methanol fixation. In comparison, little change was observed in PFA fixed cells.

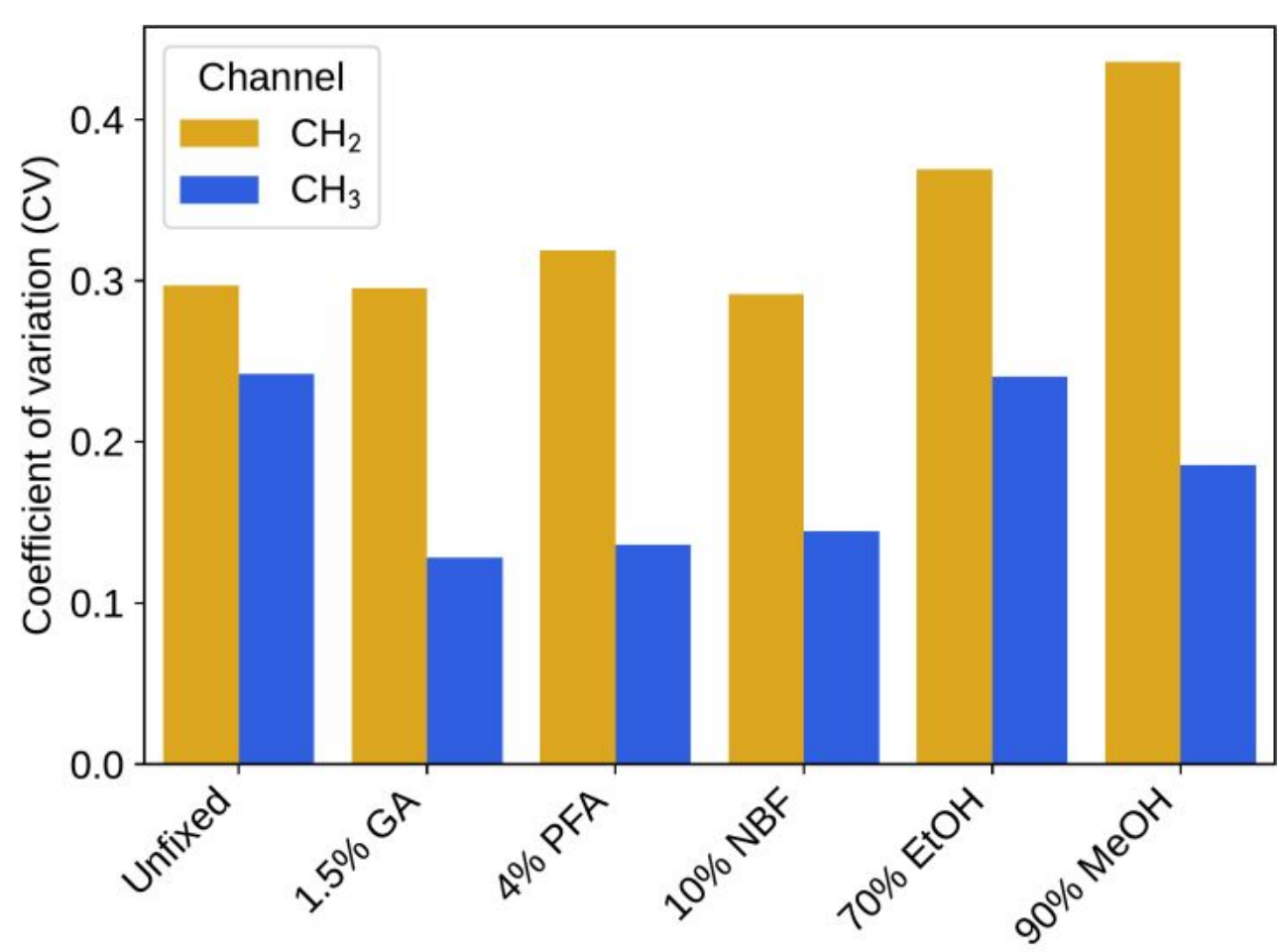

Fig. S11: Coefficient of variation of 2920 cm^{-1} (protein and lipid) and 2850 cm^{-1} (mainly lipid) SRS signal across cells. Each cell is treated as a datapoint, and per group of fixation conditions, CV is calculated as σ/μ, i.e. standard deviation of mean cellular signal of the corresponding band over aggregated mean.

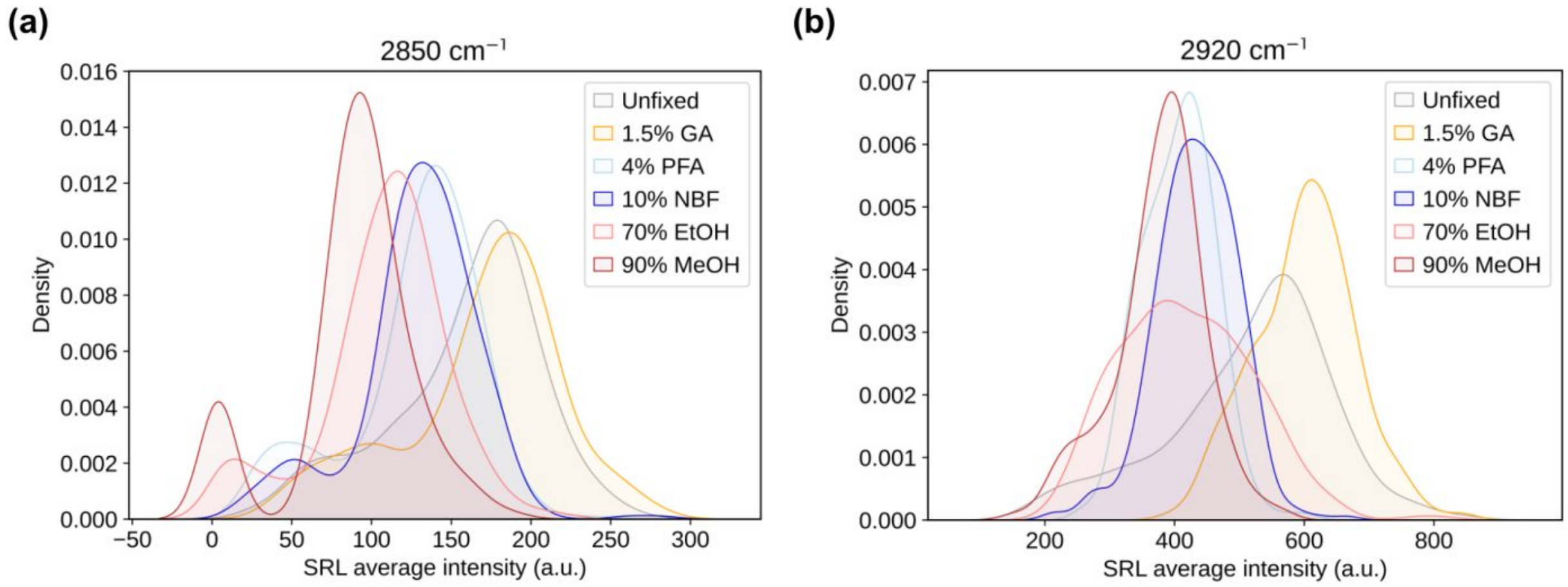

Fig. S12: Distributions of protein or lipid concentrations across cells (a) CH_2 (mainly lipid) (b) CH_3 (protein and lipid)

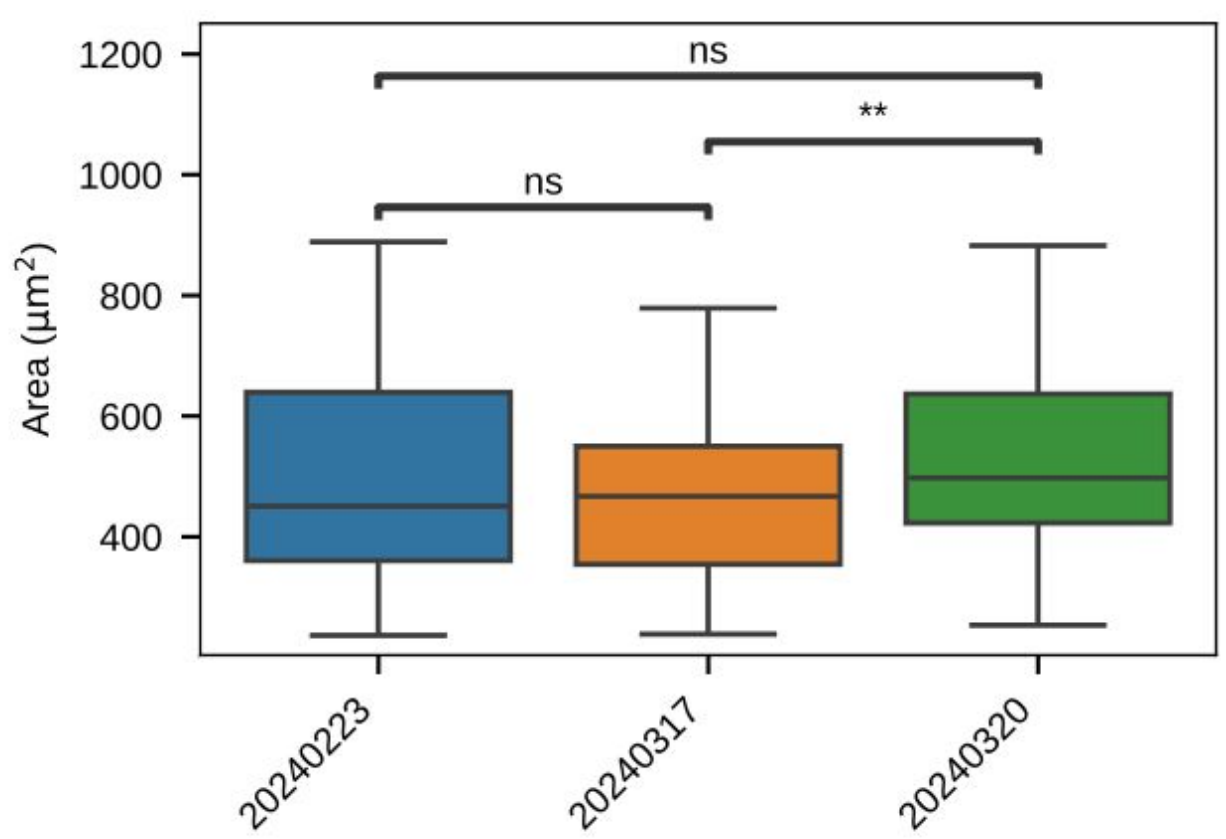

Fig. S13: Size distribution of unfixed cells measured on different days. *, **, ***, and **** indicate $p < 0.05$, 0.01, 0.001, and 0.0001 (Welch's t-test), respectively. Horizontal bars of the boxes represent maximum, 0.75, 0.5, 0.25, minimum, respectively. Outliers are determined by whether the point deviates 1.5 times the interquartile range (0.75 quartile - 0.25 quartile), from either quartile. While cell size measurements varied slightly between days, Welch's t-test revealed no statistically significant differences ($p > 0.001$, $p > 0.05$), supporting the conclusion that the measurements originate from the same underlying distribution.

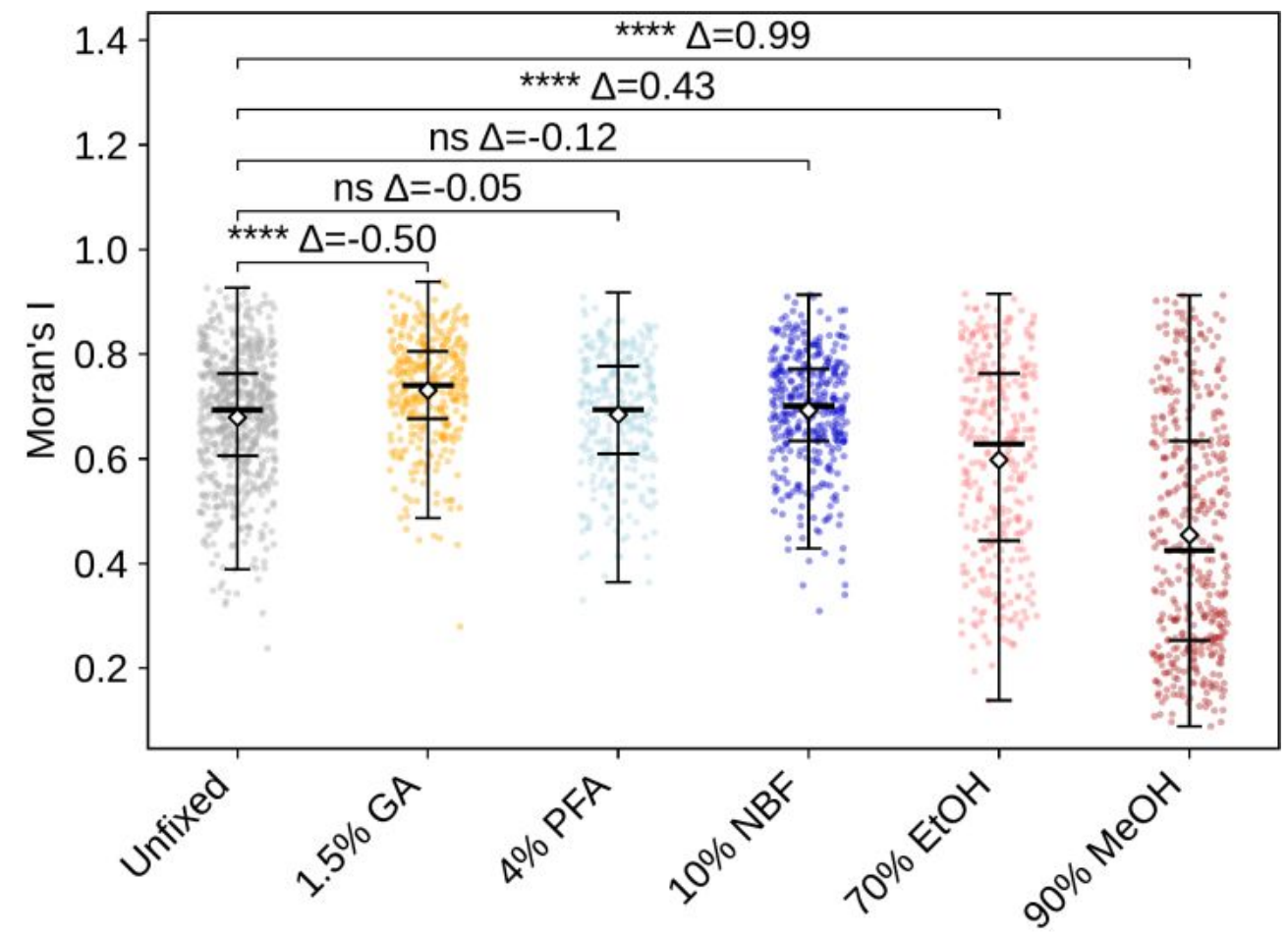

Fig. S14: Annotated strip plots of spatial autocorrelations (Moran's I). Post-hoc tests after ANOVA (Tukey's HSD) were also performed. Among all cross-linking fixatives, glutaraldehyde has a higher Moran's I (more clustered signal) that is statistically significant.

Table S1: Summary of cell counts and replicates for hyperspectral SRS imaging across fixation conditions. Biological replicates represents independent imaging experiments conducted on different days, with the exception of the unfixed control (*) which had 5 independent imaging experiments with 2 biological replicates per day.

Fixative	Biological Replicates	Number of Cells
1.5% glutaraldehyde	6	327
10% formalin	6	348
4% PFA	6	308
70% ethanol	6	331
90% methanol	6	407
unfixed	10*	604

Table S2: Summary of 2920 cm^{-1} band (protein and lipid) average intensity distributions across fixatives.

Fixative	Mean	Std	25%	50%	75%
1.5% glutaraldehyde	591	96	542	604	649
10% formalin	446	65	412	451	489
4% PFA	420	58	379	422	459
70% ethanol	429	101	352	415	493
90% methanol	388	72	349	398	433
unfixed	511	126	439	541	593

Table S3: Summary of 2850 cm^{-1} band (mainly lipid) average intensity distributions across fixatives.

Fixative	Mean	Std	25%	50%	75%
1.5% glutaraldehyde	166	52	144	178	201
10% formalin	133	39	118	137	155

4% PFA	133	42	119	141	162
70% ethanol	111	43	92	113	134
90% methanol	92	40	79	95	112
unfixed	158	46	132	168	188

Table S4: Summary of cell size distributions across fixatives. (Units in μm^2)

Fixative	Mean	Std	25%	50%	75%
1.5% glutaraldehyde	466	137	373	445	540
10% formalin	508	143	404	494	595
4% PFA	517	156	395	493	639
70% ethanol	428	153	316	396	529
90% methanol	388	137	282	363	466
unfixed	488	155	370	462	586
